# Excitation-Dependent K^+^ Sensing by Combining Photoinduced Electron Transfer and Triplet–Triplet Annihilation

**DOI:** 10.1021/acs.jpca.5c02938

**Published:** 2025-06-10

**Authors:** Hannah Tideland, Andrew J. Carrod, Yuanxin Liang, Karl Börjesson

**Affiliations:** Department of Chemistry and Molecular Biology, 3570 University of Gothenburg, Medicinaregatan 7B, 41390 Gothenburg, Sweden

## Abstract

Triplet–triplet annihilation photon upconversion (TTA-UC) combines the energy of two photons to provide one of higher energy. Detecting such high energy photons can be more selective than conventional fluorescence, because artifacts like scattering and autofluorescence do not contribute to the signal. Ions play crucial roles in biology, and quantitative in-flow sensing of ions using an all-optical readout is therefore of significant importance. Here, we assess the applicability of an anthracene-crown ether based ion sensor, which incorporates TTA-UC in combination with photoinduced electron transfer (PET). We find that these two mechanisms are compatible with each other in one functional molecule, enabling the detection of K^+^ at biologically relevant concentrations. We further find that ion binding constants differ in the electronic ground and excited states of the anthracene unit. As triplet lifetimes are on the same time scale as ion dissociation constants of crown ethers, the measured equilibrium constant depends on excitation conditions, which therefore must be taken into account in the analysis. Lastly, we built a microfluidic device in order to demonstrate how in-flow ion sensing could be conducted and achieve scattering free upconversion signals and predictable binding constants. This work examines TTA-UC-based ion sensing from a mechanistic to an application perspective and provides a step toward quantitative all-optical sensing of biologically relevant ions in flow.

## Graphical abstract

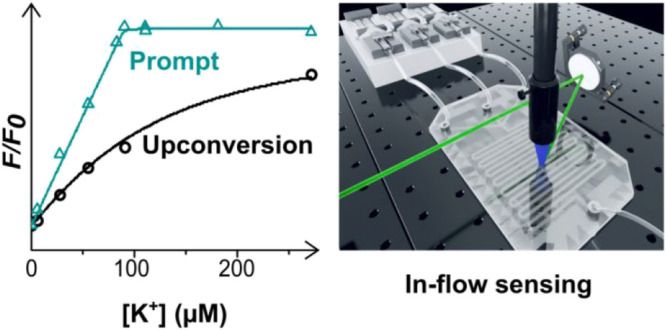

## Introduction

Ions play crucial roles in many biochemical processes such as osmosis, signal transmission and metabolism. Potassium and sodium are the two dominant ions inside the human cell and in the extracellular matrix, respectively.^1^ Together, these cations regulate vital cell functions such as fluid and electrolyte balance, blood pressure, neurotransmitter signaling, and the cell’s action potential.^1,2^ The concentrations of these ions in the body are normally maintained within a narrow range and even small deficiencies/excesses can lead to a disruption of normal bodily functions. The link between physiological ion concentrations and disease has prompted the development of several ion sensing methods in vitro. Common methods in use today are atomic absorption and emission spectroscopy, inductively coupled plasma mass spectroscopy (ICP-MS) and electroanalytical techniques.^1,2^ However, these methods generally require expensive instrumentation, complex sample preparation, and have limitations in the speed and resolution of detection.

In recent decades fluorescent probes have emerged as an alternative because of their generally high sensitivity, selectivity, versatility, and relative speed and ease of the analysis. Detection limits are typically below μM down to a few nM.^3^ Ion-sensing fluorescent probes are most often organic molecules, consisting of a metal complexing moiety, usually called the receptor or ionophore, a fluorophore responsible for the emission, and often a covalent spacer between these two.^1,2^ A fluorescence change in response to ion complexation can be achieved through several quenching/enhancement mechanisms that cause the relaxation pathway to be altered with analyte binding. Quenching mechanisms can be broadly divided into (1) ratiometric mechanisms where the ratio of two or more emission bands change with analyte complexation, e.g., Förster resonance energy transfer and intramolecular charge transfer, and (2) on–off mechanisms, where a dark state quenches the emission in absence of analyte, e.g., in twisted intramolecular charge transfer and photoinduced electron transfer.^4–8^ In photoinduced electron transfer (PET), an electron is transferred from a frontier orbital of the donor to the acceptor, resulting in the formation of a dark charge-separated state (CSS). Binding to an analyte such as a cation tends to make the charge separation much less favorable, resulting in a recovery of emission.

Despite the clear benefits of fluorescence sensing, many efficient fluorophores absorb in the UV or blue light range causing issues. Especially for in vitro analysis, where autofluorescence and scattering add noise and artifacts. Consequently, it would be useful to take advantage of an anti-Stokes shift process, where the wavelength of emission is shorter than that of absorption. The most common techniques to achieve such are two-photon absorption,^9–12^ rare-earth upconversion,^13,14^ and triplet–triplet annihilation upconversion (TTA-UC). Compared to the other processes mentioned, TTA-UC requires relatively low excitation intensity, gives an overall higher quantum efficiency than rare-earth upconversion, and allows tuning the excitation and emission wavelengths by combining and chemically modifying the sensitizer and annihilator components of the system.^15,16^ The phenomenon was discovered in the 1960s when investigating delayed fluorescence in anthracene derivatives.^17,18^ It has come into the spotlight as an independent research field in the mid-2000s, due to its potential for utilizing longer wavelengths of the solar energy spectrum in solar cells^19^ as well as photocatalysis,^20–23^ and biosensing.^24–29^

TTA-UC is a photophysical process that through a series of steps produces a higher energy emission than that of the absorption. The process requires sensitizer, S, and annihilator, A, molecules, and the mechanism is outlined in Figure 1 (see Supplementary Section 3 for a detailed description). The quantum yield of the upconversion process is the product of the quantum yields of the intermediate photophysical processes.

$$\Phi_{\mathrm{UC}}=\Phi_{F}\Phi_{\mathrm{TET}}\Phi_{\mathrm{TTA}}\Phi_{\exp}$$ 1

where Φ_F_, Φ_TET_, and Φ_TTA_ are the quantum yields of fluorescence, triplet energy transfer, and triplet–triplet annihilation, respectively. The last factor, Φ_exp_, signifies the outcoupling losses such as the inner filter effect and scattering.^30^ Note that the triplet–triplet annihilation quantum yield, Φ_TTA_, has a maximum value of 0.5. This factor includes the fraction of triplet pairs that form the excited singlet state, known as the spin-statistical factor.

**Figure 1:**
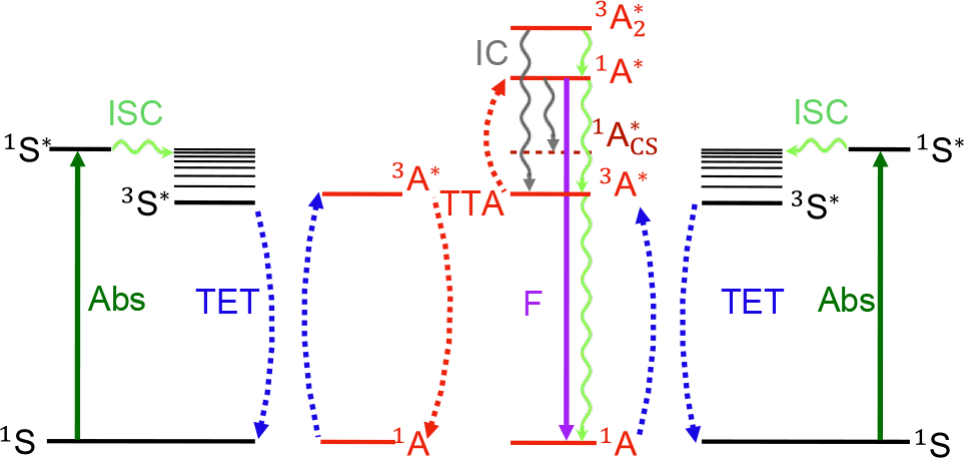
Simplified Jablonski diagram of the TTA-UC process with the two molecular components, the sensitizer (S) and the annihilator (A). Left superscripts 1 and 3 denote singlet and triplet states, respectively, and the right superscript star (*) denotes an excited state. The photophysical processes depicted are absorption (abs), intersystem crossing (ISC), triplet energy transfer (TET), triplet–triplet annihilation (TTA), internal conversion (IC), and fluorescence (F).

The literature on TTA-UC-based ion sensing is currently quite scarce. A TTA-UC and PET-based sensor selective for Mg^2+^ was developed for use in DCM (anthracene -crown ether dyad),^27^ and more recently for Ca^2+^ in methanol DCM (perylene based).^29^ Hg^2+^ sensors partly based on TTA-UC with nM limits of detection have also been reported.^25,28^ Furthermore, Jewell et al., utilized the common sensitizer-annihilator pair PtOEP and 9,10-diphenylanthracene embedded in a polymer together with a K^+^ binding dye for in vivo imaging.^26^ However, studies so far have not focused on the mechanisms affecting the sensitivity and dynamic range of the sensors. TTA-UC is a multistep process where the rate and quantum yield of each step may be different in presence or absence of the analyte. The molecular weight, the geometry and energy of excited triplet states, and the potential presence of additional charge-separated states are all factors that may influence the sensitivity. Furthermore, because of the long lifetime of triplets, analytes may associate or dissociate not only with the ground state but also with ^3^A*. Thus, the sensitivity might depend on the nature of excitation. To the best of our knowledge, these questions have not been investigated directly in previous literature on TTA-UC sensing.

Here, we systematically study the recovery of prompt as well as upconverted fluorescence in an anthracene-based potassium sensor. We particularly address the influence of potential triplet state quenching by the CSS, the triplet geometry, and the association and dissociation kinetics, on the sensitivity of TTA-UC sensing. We further discuss how TTA-UC based PET sensing can be tuned for optimal sensitivity or analyte response range. Finally, to put our findings to practical use, we develop a microfluidic setup appropriate for TTA-UC sensing. The facile setup allows TTA-UC measurements in continuous flow and could thus relatively straightforwardly be incorporated in a clinical lab. We hope that the conclusions presented herein will facilitate the use of upconversion in sensing applications, as anti-Stokes detection provides selectivity even in autofluorescent media.

## Results and Discussion

### Molecular Design Considerations

The design of the upconversion based ion sensors was motivated by acquiring an appropriate sensitivity, selectivity, and dynamical range. For sensors utilizing PET, the CSS should quench the fluorescence when no analyte is present while being energetically unreachable when bound to the analyte ion. The difference in quenching between the free and bound states constitute the dynamic range and thus the sensitivity. Upconversion systems based on anthracene were chosen in this study. Anthracene is probably the most well studied upconversion system to date and is therefore particularly suitable in application-based studies. Anthracene has a relatively high photostability, high electron accepting ability, and is easily modified at the 9- and 10-positions,^31–33^ i.e., along the direction of the S_1_ → S_0_ transition dipole moment.^34–36^ Two anthracene derivates having crown ethers, attached via amine or amide functionalities at position 9, were synthesized (Supplementary Section 2 and Figures S1–S5). The amine and amide functionalities have electron donating capabilities, providing the system with a CSS with an energy of the same order of magnitude as the first excited singlet state of anthracene. Crown ethers are well-known to selectively bind ions based on the matching size and shape of ion and binding cavity. They provide sufficient selectivity for environments where the analyte is more concentrated than other ions, such as potassium in human cells.^37–39^ Furthermore, crown ethers are sufficiently small to not influence the diffusion limiting process of TTA-UC. In this study, crown ethers having a size suitable to bind K^+^ and secondarily Na^+^ were used.

Figure 2 shows the structure of the two molecular sensors. Molecule **1** is previously not studied, while molecule **2** has been used as a fluorescent K^+^ sensor, with a measured rate of excited state charge separation of 10^–10^ s^–1^.^40^ The difference in electron density between the first excited and ground singlet states of the molecules were calculated with TD-DFT at the B3LYP/6-31+G­(d,p) level using the Gaussian 16 suite.^41–45^ This calculation was performed for both the free and K^+^ bound molecules. The calculations for the unbound molecules predict a charge separation of the S_1_ state (Figure 2a,b). This is evident by anthracene having an increase in electron density (yellow) whereas the amine/benzoquinone-amide functionalities have a decrease in electron density (blue). The calculations predict that the lowest bright singlet is over 1.2 eV higher than the CSS in both molecules (Figure 2c,d). However, when the crown ether is bound to K^+^ the situation alters dramatically (Figure 2e–g). In this form, the CSS energy increases by over 1 eV (Figure 2a,b vs e), while the energy of the locally excited state remains essentially the same as in the free form. There is also a significant distortion of the crown ether in the relaxed CSS of **1**, indicating a change in the binding properties. Furthermore, the adiabatic and vertical energies of the locally excited state are lower than that of the CSS in the K^+^ complex (Table S1). In the **2**-K^+^ complex, the relaxed locally excited state is also the lowest energy singlet (Figure 2g). The CSS is significantly higher in energy and corresponds to the LUMO ← HOMO-2 transition (3rd vertical excited state, see Table S1 and Figure S6). It does not form a stable minimum on the potential energy surface. These conclusions are in agreement with previous computational studies on the compound. The oscillator strengths of the relaxed excited singlets in both free and K^+^ complexing forms indicate that the CSS is dark, while the locally excited state is emissive (Table S1).^46,47^ Overall, the calculations point to a strong destabilization of the CSS in the K^+^ complex of both molecules. These results are in line with chemical intuition, i.e., complexation by the lone pair of N to K^+^, results in N becoming a much weaker electron donor. Consequently, the CSS increases radically in energy and the anthracene localized state becomes the lowest. Similar results were found for the Na^+^ ion (Table S1, Figures S6 and S7).

**Figure 2:**
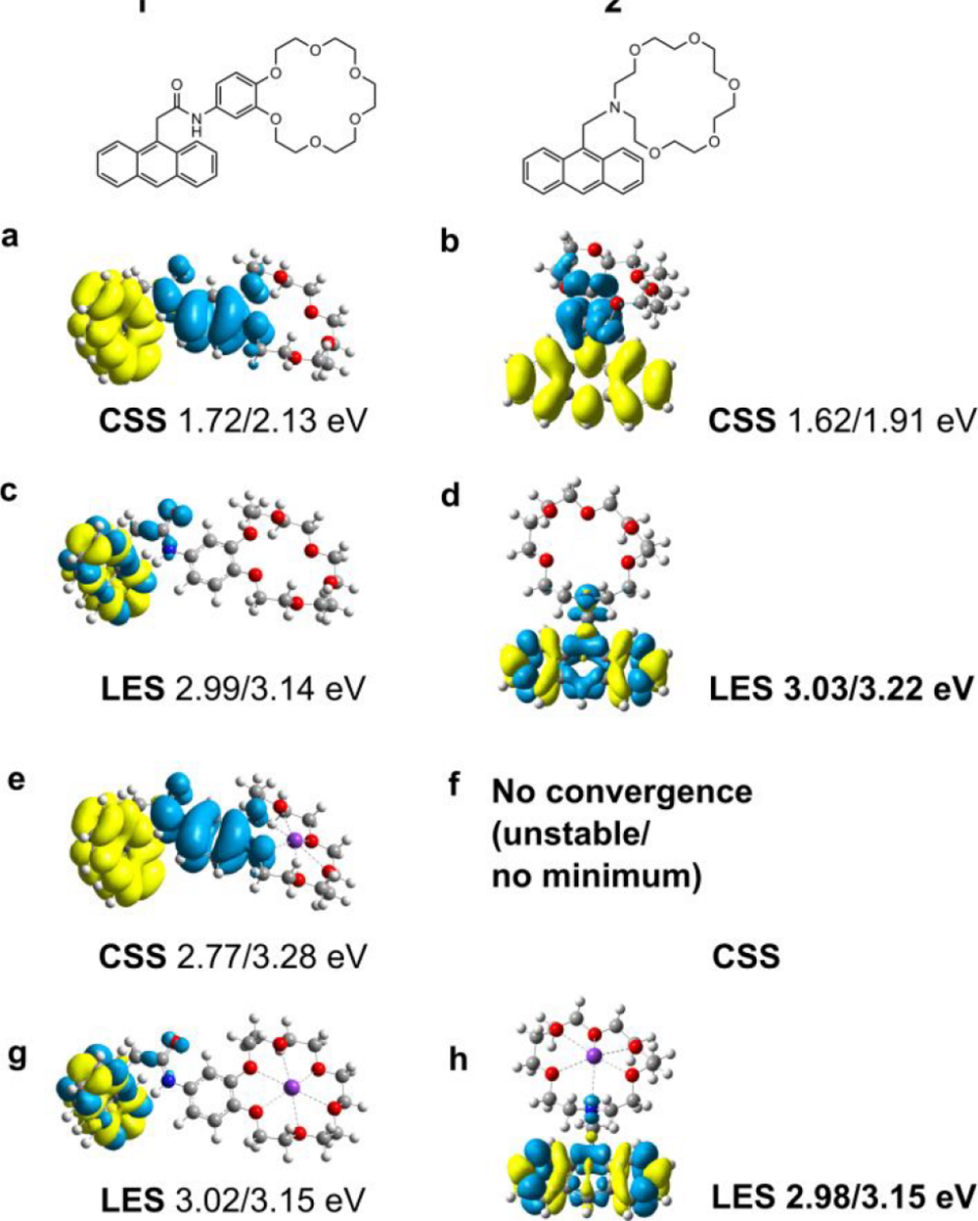
Chemical structure of molecules **1** and **2**, electron density differences (isovalue 0.005) and relaxed/adiabatic transition energies. These states correspond to the CSS (a,b,e,f) and locally excited (LE) lowest singlet (c,d,g,h) in free (a–d) and K^+^ bound (e–h) forms. The CSS of **2** (f) did not form a stable minimum on the excited singlet surface and thus not included here. Calculations were done at the TDA-B3LYP/6-31+G­(d,p) level. Blue fields indicate electron deficiency while yellow depicts electron surplus.

We note that limited inclusion of electron correlation of common DFT functionals like B3LYP cause the excited to ground state transition energies to be underestimated.^48^ Nevertheless, the error can be expected to be similar for the CSS and local excited state since calculated values do not show a strong dependence on the polarity of the system. Furthermore, functionals with a similar amount of exact exchange (20–25%) are routinely used in the literature of PET.^46,47^

### Prompt Photophysical Properties

To assess the capacity of the molecules as TTA-based sensors, the prompt photophysical properties and its effect by ion binding was first evaluated. This was done in three different solvent systems of varying polarity: dichloromethane (DCM), methanol and a 3:7 water:methanol mixture. The photophysical properties are summarized in Table 1 and outlined below. The absorbance and emission spectra of the S_1_–S_0_ transition of both molecules in all solvents were similar to that of anthracene (Figure S8) indicating a localization of the S_1_ state on the anthryl moiety. The S_2_–S_0_ transition of **1** is partly visible, indicating a lowering of the S_2_ state compared to anthracene.^49^ The maxima of the 0–0 transitions were located at 386 and 389 nm for **1** and **2**, respectively. The extinction coefficients are in the 8500–10,000 M^–1^cm^–1^ range for **1** (Figure S9), in line with the literature value of anthracene (values of 8.8 × 10^3^ and 8.5 × 10^3^ M^–1^cm^–1^ have been reported in cyclohexane and acetonitrile, respectively).^50,51^ The value for molecule **2** is also similar in DCM. The fluorescence quantum yields were 3–5% for **1** and 6% for **2** with higher values in the less polar DCM. The emission quantum yield is at least 7 times lower than for anthracene and 9-methylanthracene,^50,52–54^ which is indicative of a quenching process being active. As quantum mechanical calculations predict the charge separated state to be lower in energy than the localized S_1_ state (Figure 2), we attribute this low fluorescence quantum yield to a PET process. Furthermore, the energy of the CSS decreases with solvent polarity, resulting in a larger driving force with solvent polarity, explaining the lower fluorescence quantum yield in the more polar solvents.

**Table 1:** Prompt Photophysical Parameters, Including Fluorescence Quantum Yields, Φ_F_ and Molar Extinction Coefficients at the Absorption Maxima ε_max_ in the Different Solvent Systems Tested^a^

molecule	solvent	Φ_F_ (%)	ε_max_ (10^3^ M^–1^ cm^–1^)
**1**	DCM	5.3	8.5 ± 1 (387 nm)
	MeOH	4.7	9.4 ± 1 (386 nm)
	3:7 H_2_O:MeOH	3.8	10.2 ± 0.1 (387 nm)
**2**	DCM	6.4	9.0 (389 nm)

a 2 was not tested in MeOH and 3:7 H_2_O:MeOH due to insufficient solubility.

Titrations with K^+^, Na^+^, and NH_4_^+^ in the three solvent systems were performed to evaluate the ion selectivity, sensitivity, and dynamical range of **1**. Titrations using molecule **2** are not reported due to limited solubility. Figure 3a shows a representative titration, displaying the absorbance and fluorescence of **1** in methanol with an increasing amount of potassium acetate (KOAc). The absorbance was unaffected by salt in the relevant wavelength region. However, although the spectral envelope was unaffected by increasing salt concentration, the fluorescence intensity increased about 3 times.

**Figure 3:**
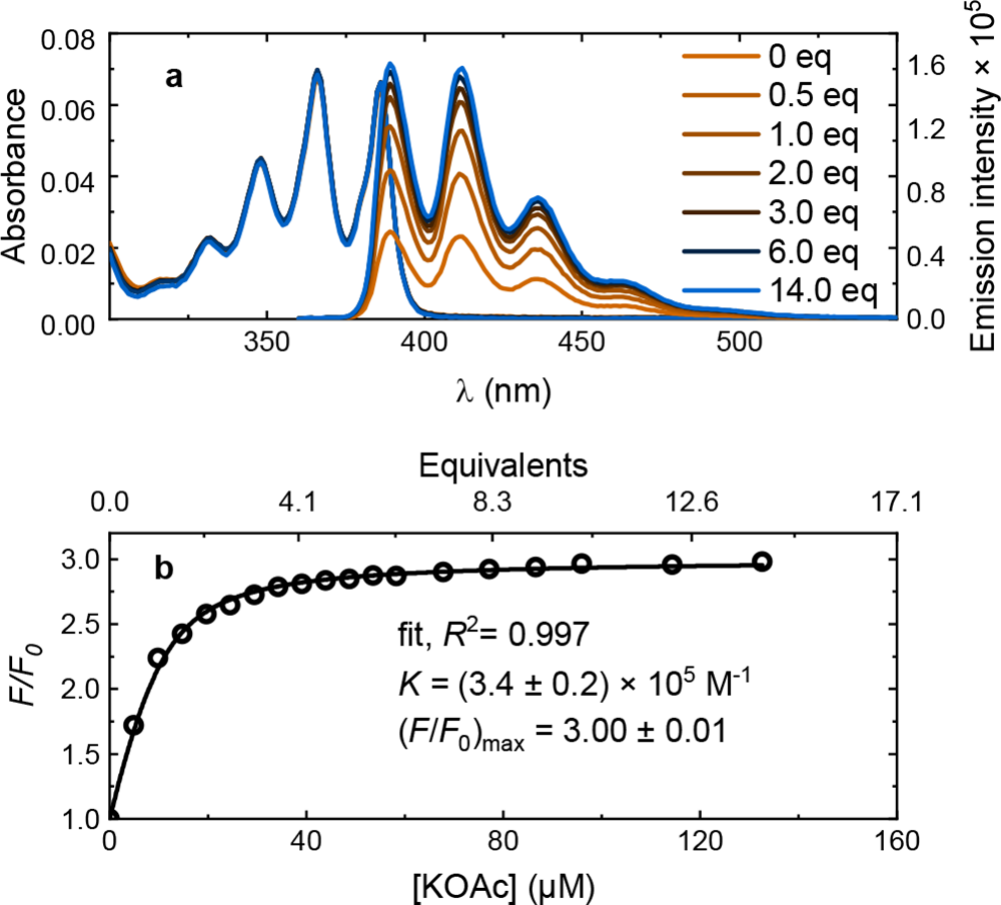
Fluorescence titration data for sensor **1** in methanol with KOAc addition including (a) absorbance, and (b) relative (integrated) fluorescence, *F/F*_0_, against KOAc concentration together with a fit to eq 2 using a 1:1 static binding model (black solid curve).

The fluorescence spectra were then integrated and compared to the value received at zero salt concentration, *F*_0_, resulting in a relative fluorescence intensity with salt concentration, *F*/*F*_0_. By fitting *F*/*F*_0_ to the salt concentration, the ion selectivity, sensitivity, and dynamical range could be quantified using a 1:1 static binding model (see Supplementary Section 1 for derivation).^3^

$$\frac{F}{F_{0}}=1+\left(\frac{0.5([H{]}_{0}+[G{]}_{0}+1/K)-\sqrt{0.25{\left([H{]}_{0}+[G{]}_{0}+\frac{1}{K}\right)}^{2}-[H{]}_{0}[G{]}_{0}}}{[H{]}_{0}}\right)\times \left({\left(\frac{F}{F_{0}}\right)}_{\max}-1\right)$$ 2

Here, (*F*/*F*_0_)_max_ is the asymptotic value of *F*/*F*_0_ at high salt concentrations, [H]_0_ is the total concentration of the host (here molecule **1**), [G]_0_ is the added guest (cation) concentration, and *K* is defined as the equilibrium binding constant:

$$K=\frac{\left[\mathrm{HG}\right]}{\left[H][G\right]}$$ 3

where [HG] and [H] are the respective concentrations of the host–guest complex and host. Hence, the magnitude of *K* indicates the sensitivity of binding between the molecule and the cation. A much higher *K* for one cation compared to another is a necessary but not sufficient condition for selectivity. The maximal dynamic range, i.e., the maximum difference in fluorescence with and without bound ions, corresponds to (*F*/*F*_0_)_max_.

Figure 3b depicts eq 2 fitted to the KOAc titration of **1** in methanol. *K* is on the order of 10^5^ M^–1^. Although data is not available for 4′-aminobenzo-18-crown-6 or fluorescent derivatives, crown ethers of similar size have binding constants on the same the order of magnitude for K^+^.^55–57^ Figure 4 (and Figures S10–S17) shows *K* and (*F*/*F*_0_)_max_ values for all ions and solvents. The sensitivity was largest in the lowest polarity solvent, DCM, with a *K* values of 3.5 × 10^7^ M^–1^, 2 orders of magnitude higher than in methanol and 3 orders of magnitude above that of the 3:7 water:methanol mixture.

**Figure 4:**
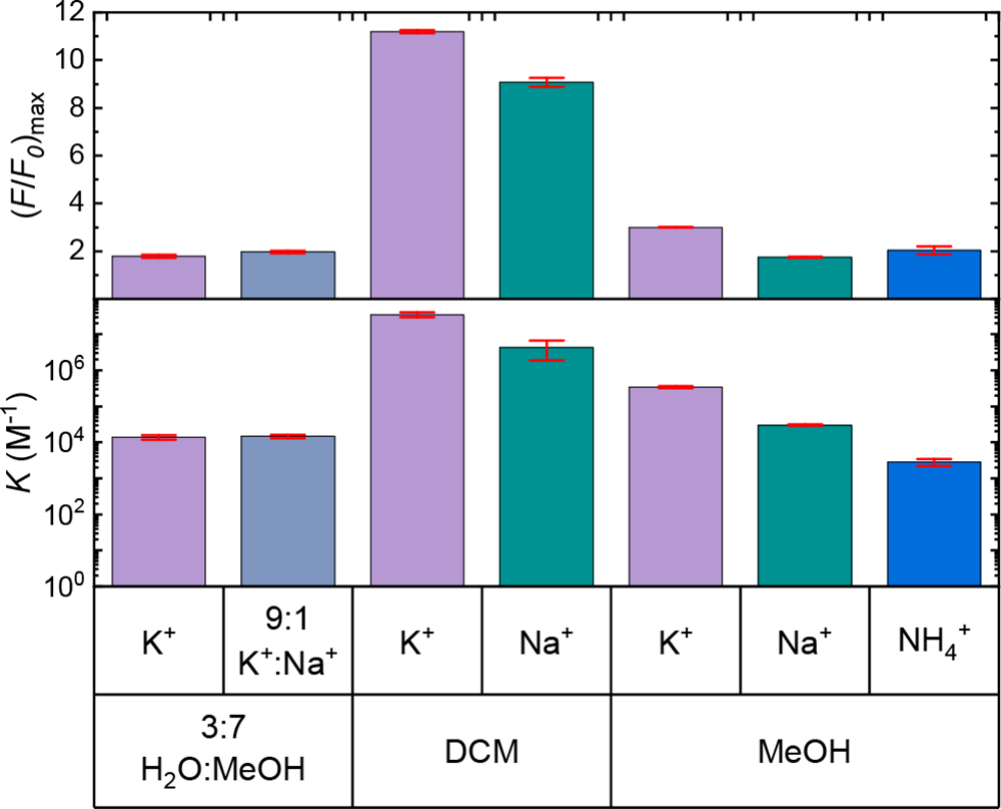
Summary of fluorescence titrations for sensor **1** in the three solvent systems in terms of binding constant, Κ, (logarithmic scale) and maximum relative fluorescence, (*F*/*F*_0_)_max_. Error bars represent the standard error. Counterions were acetate in MeOH and 3:7 H_2_O/MeOH, and perchlorate in DCM. Different salts were used in the solvents to maximize solubility.

Partial selectivity for K^+^ over Na^+^ was observed, with *K* values for K^+^ being 8 and 11 times higher than for Na^+^ in DCM and methanol, respectively. Furthermore, the *K* value for K^+^ in methanol is over 120 times higher than for NH_4_^+^. In addition, in the water:methanol mixture, NaOAc and NH_4_OAc titrations did not show any significant change in fluorescence (Figures S16 and S17). To test whether this lack of fluorescence enhancement result is due to lack of binding, at least at biologically relevant concentrations, titrations were also performed with a concentration ratio of 9:1 KOAc:NaOAc (Figure S15). This provides an upper estimate of the fraction of Na^+^ for noncancerous human cells.^58^ The titration resulted in *K* values within standard error of those for K^+^, indicating that the Na^+^ ions do not bind significantly to the crown ether cavity in this more polar solvent. Thus, sensor **1** appears to bind to K^+^ without any interference from Na^+^. The higher selectivity in the more polar solvent may result from Na^+^ having a much greater affinity for the bulk solvent, than the crown ether cavity. Further studies are required to elucidate whether this is due to the high polarity of the bulk solvent or specific ion–solvent and crown ether–solvent interactions.

The dynamic range showed a clear dependence on solvent polarity. The largest dynamic range was found for both molecules in the lowest polarity solvent, DCM, indicated by (*F*/*F*_0_)_max_ values of 11 and 10 with KClO_4_ and NaClO_4_, respectively. This corresponds to a maximum Φ_F_ above 50%, suggesting that PET quenching was energetically unfavorable in the bound state. This is much larger than in methanol, where an (*F*/*F*_0_)_max_ value of 3.0 was determined for KOAc, while values for NaOAc and NH_4_OAc were 1.7 and 2.1, respectively. The highest value of 3.0 in methanol corresponds to a maximum fluorescence quantum yield of 14%. This is about half the value of that of unsubstituted anthracene in ethanol and acetonitrile,^52–54^ which have polarities just below and above that of methanol. 9-substituted anthracenes with rigid and electron-donating substituents tend to have an even higher fluorescence quantum yield.^59,60^ Thus, our results indicate that the CSS is still energetically reachable through thermal activation in methanol. Titrations in the most polar solvent system, i.e., the water:methanol mixture resulted in the lowest (*F*/*F*_0_)_max_ value of 2.1 for both KOAc and the 9:1 KOAc:NaOAc mixture.

In summary, our results show that the sensitivity and dynamic range decreases with solvent polarity. Trends are in line with Rehm–Weller theory and chemical intuition (Supplementary Section 4), i.e., CSS is stabilized, and ion binding is less favorable in more polar media. The selectivity does not follow a clear trend in terms of bulk solvent properties. However, a particularly high sensitivity was found in the water:methanol mixture, which is promising for the development of crown ether-based ion sensors.

### Triplet Energy Transfer

Having discussed the merits of the molecules as cation binders, we now turn our attention to their properties as upconversion annihilators. TTA-UC relies on three events (Figure 1). The second one is triplet energy transfer from the sensitizer to the annihilator, and it was evaluated by a Stern–Volmer quenching analysis. Since TET is a form of dynamic quenching, the phosphorescence of the sensitizer follows the relationship:

$$\frac{I_{0}}{I}=\frac{\tau_{0}}{\tau }=1+k_{\mathrm{TET}}\tau_{0}[A]$$ 4

$$\Phi_{\mathrm{TET}}=\frac{k_{\mathrm{TET}}[A]}{k_{\mathrm{TET}}[A]+k_{0}}=1-\frac{I}{I_{0}}$$ 5

where *I* and *I*_0_ are the phosphorescence intensities of the sensitizer with and without the annihilator, and τ and τ_0_ are the respective lifetimes. *k*_TET_ is the rate constant of TET, *k*_*0*_ is the rate constant of sensitizer phosphorescence, and [A] is the annihilator concentration. Figure 5a (see Figure S18 for individual spectra) shows the relative maximum intensity of 5 μM PtOEP in DCM with increasing concentrations of molecules **1** or **2**, as well as the corresponding quantum yields of TET (Φ_TET_). Fits to eq 4 gave rate constants of 3.5 × 10^9^ and 4.2 × 10^9^ s^–1^ for **1** and **2**, respectively. These data indicate that TET is nearly diffusion-limited for both annihilators. The high efficiency of TET can be rationalized by the lower triplet energy of anthracene derivatives, ≈1.8 eV,^61–63^ compared to the sensitizer. The slightly lower efficiency for **1** can be explained by a higher molecular weight and thus slower diffusion.

**Figure 5:**
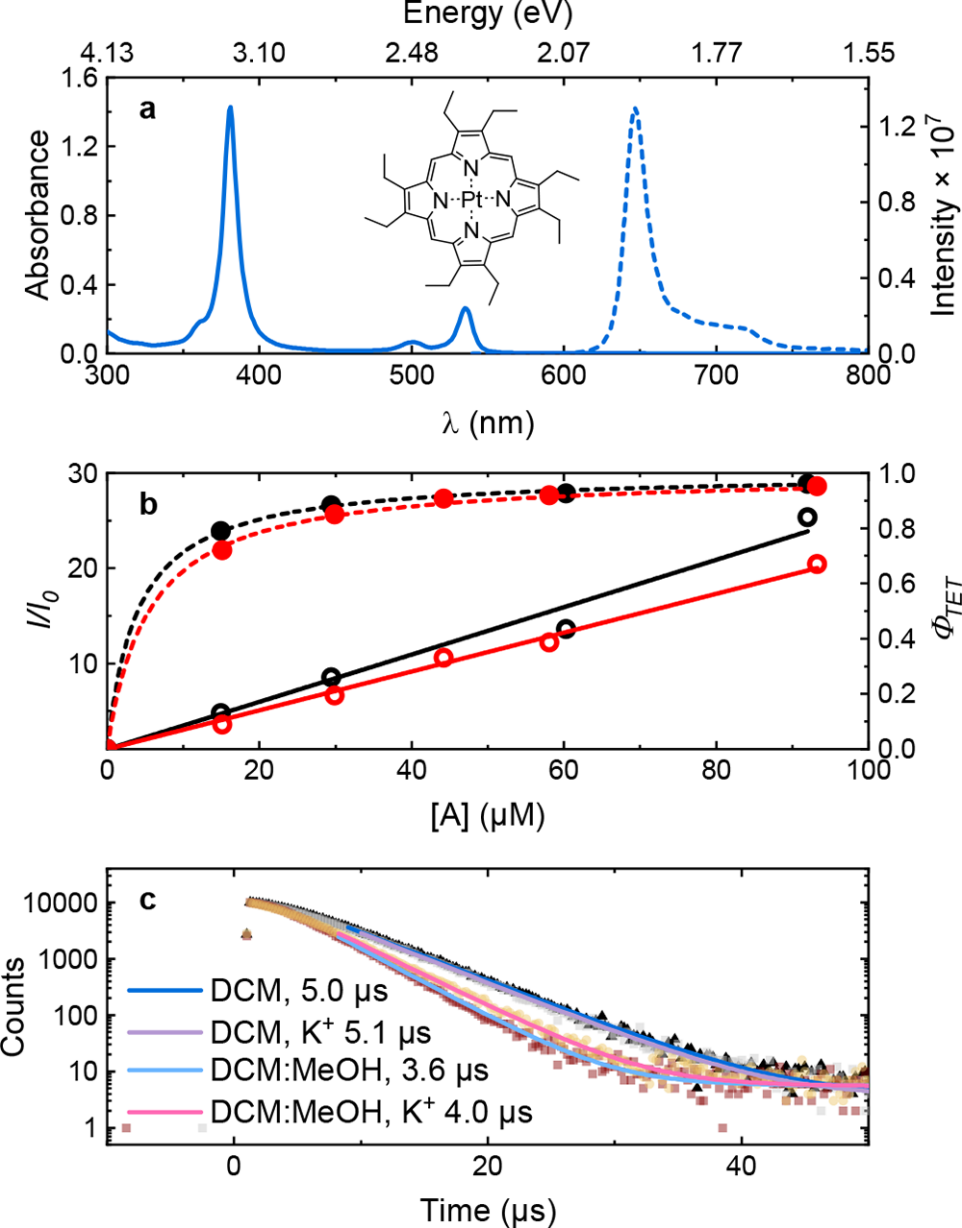
(a) Molecular structure, absorbance (solid line) and emission (dashed) of 5 μM PtOEP excited at 532 nm. (b) Stern–Volmer quenching (open circles) fit to eq 4 (solid lines) and quantum yields of TET (open circles) fit to eq 5 (dashed) taken from the emission intensity at 650 nm, for molecules **1** (red) and **2** (black) in DCM. (c) Sensitizer emission decay fit to a double-exponential with sample and initial sensitizer lifetimes in the legend.

The sensitizer has a monoexponential phosphorescence decay with a lifetime, τ_TS_^0^ (Table 2 and Figure S19), in the range of literature values (50 ± 20 and 71 μs in CHCl_3_ and DCM respectively),^64,65^ indicating that oxygen had been efficiently removed. In the presence of **1**, the lifetime, τ_TS_, is more than 10 times shorter compared to τ_TS_^0^ (Figure 5c). Furthermore, τ_*TS*_ is 10% longer in a KOAc-containing solution than in the salt free DCM:methanol solution (Figure 5c and Table 2). This corresponds to a reduction in Φ_TET_ (eq 5) from 0.942 to 0.935. The reduction is most likely due to the slower diffusion of **1**-K^+^ compared to **1**. This conclusion is supported by transient absorption measurements showing a slightly lower ^3^A* concentration in the presence of KOAc at the start of its decay (Figures S20–S23). The effect is not observed in pure DCM, likely because of the lower solubility of KClO_4_ and low solubility and dissociation of K^+^ salts in low polarity solvents in general. Thus, the presence of ions does not affect the TET process except for a small reduction in the rate of diffusion of **1**.

**Table 2:** TTA-UC Parameters for 1 Used as Annihilator at Concentrations of 45–90 μM with PtOEP as the Sensitizer (2.5-5 μM)^a^

parameter	DCM	1:1 DCM:methanol
τ_ΤΑ_/ms	0.90 ± 0.07	0.93 ± 0.15
τ_ΤΑ_/μs (K^+^)	0.70 ± 0.02	0.77 ± 0.08
τ_ΤS_/μs	5.0	3.6
τ_ΤS_/μs (K^+^)	5.1	4.0
τ_TS_^0^/μs	59	62
*k*_TTA_/(10^9^ M^–1^ s^–1^)	2.5 ± 0.6	2.2 ± 1.5
*k*_TTA_/(10^9^ M^–1^ s^–1^) (K^+^)	2.4 ± 0.1	1.4 ± 0.2
Φ_UC_	0.0045	0.0042
*I*_th_/(W cm^–2^)	0.88	

a K^+^ was added in the form of KClO_4_ in DCM and KOAc in 1:1 DCM:methanol, respectively. Different salts were used in the two solvents to maximize the solubility.

### Upconversion of Molecule **1**

Having determined that charge separation strongly reduces the quantum yield of fluorescence but not that of TET, we now focus on the remaining photophysical process, that of TTA. The photophysical parameters of upconversion for **1** are summarized in Figure 6 and Table 2. The steady state upconversion spectrum of **1**, with PtOEP as the sensitizer, is given in Figure 6a. The upconverted fluorescence can be seen in the 390–520 nm wavelength region, whereas the band at 650 nm corresponds to the sensitizer phosphorescence. A small inner filter effect is observed for the annihilator emission. This is due to the high concentration (90 μM) of **1** needed to achieve an efficient TET.

**Figure 6:**
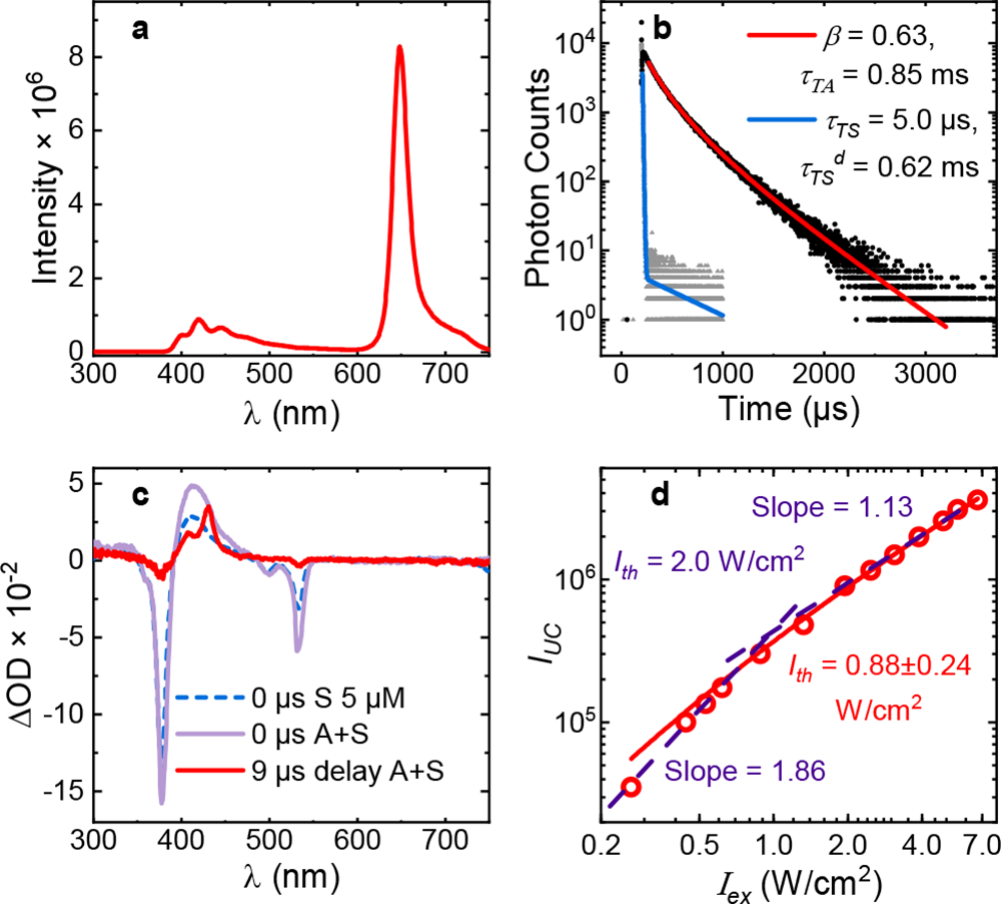
Upconversion of **1** as the annihilator and PtOEP as the sensitizer in DCM. (a) Upconversion spectrum. (b) Time-resolved emission of the annihilator (415 nm, black dots) fitted to eq 6 (red), and the sensitizer (647 nm, gray dots), fitted to a biexponential decay (blue). (c) Nanosecond transient absorption spectra at 0 and 9 μs delay (purple and red, respectively), and the sensitizer spectrum at 0 μs delay (blue dashed). (d) Upconversion intensity against excitation intensity, fitted to eq 8 (red), as well as power functions (purple dashed).

Time resolved emission reveals an initial decay of sensitizer emission at the same time scale as a rise of the upconversion signal (Figures 6b and S24–S35). There is also a much weaker slow decay component of the sensitizer (<1% amplitude) attributable to reabsorption of the upconverted emission or annihilator to sensitizer FRET (Figures S36–S40).

The time-dependent intensity decay of ^3^A* after laser excitation (Figure 6b, black dots, Figures S24–S35) was fitted to the following equation:^66^

I(t)∝[A3*]2=([A3*]01−βexp(t/τTA)−β)2 6

where τ_ΤΑ_ is the triplet lifetime of the annihilator and the β parameter corresponds to the fraction of ^3^A* that decay through TTA (as opposed to any other processes, including phosphorescence, ISC and PET). Specifically β is defined as^67^

β=2kTTA[A3*]01/τTA+2kTTA[A3*]0 7

The model assumes no second order processes except for TTA of the annihilator molecule. The resulting τ_ΤΑ_, values were approximately 0.9 ms in both DCM and a 1:1 DCM:methanol mixture without salt. The lifetimes are in the range typically reported for deaerated solutions of anthracene and 9- and 10-substituted anthracene derivatives at this concentration (10^–4^–10^–2^ s with larger values for 9,10-substituted anthracenes with bulky substituents).^68–70^ Furthermore, the presence of an excess of K^+^ caused a reduction in τ_TA_ by 0.2–0.3 ms in both solvents (Table 2). There are two notable conclusions from this result. First, an increase in τ_TA_ would be expected with ion complexation if ^3^A* was significantly quenched by a CSS. Hence, quenching by the CSS is unfavorable (for both CSS on the singlet and triplet surfaces). This conclusion is supported by TD-DFT calculations (Figure S41 and Table S1), where the lowest triplet is essentially equal in energy in both free and K^+^-bound forms and in both cases corresponds to the localized π–π* transition. In addition, the energy of ^3^A* is at least 0.4 eV lower than that of the CSS, making intersystem crossing to the latter highly unlikely. Second, the reduction in τ_TA_ cannot be attributed to a faster TTA (vide infra), hence the nonradiative decay rate must be higher. The reason for this is not clear.

The rate, *k*_TTA_, was determined from the emission decay in combination with the transient absorption spectrum (Figure 6b,c). The initial triplet concentration, [^3^A*]_0_ was calculated from the transient optical density at 427–430 nm at a delay of 9 μs (Figure 6c), corresponding to the maximal value of the excited state absorption of ^3^A*. The molar extinction coefficient of the triplet state was approximated by that of anthracene in cyclohexane (85,700 M^–1^cm^–1^).^71^ The estimate is likely to be reasonable given the similarity of the ground state molar extinction coefficient of **1** to that of anthracene.

The value of *k*_TTA_ was determined to (2.5 ± 0.6) × 10^9^ M^–1^ s^–1^ in DCM and (2.2 ± 1.5) × 10^9^ M^–1^ s^–1^ in DCM:methanol. A small reduction could be seen in KOAc-saturated conditions, (1.4 ± 0.2) × 10^9^ M^–1^ s^–1^, most likely due to a reduced rate of diffusion. The rate constants are also somewhat lower than for anthracene,^72,73^ 9,10-diphenylanthracene,^66,74^ and several other anthracene-based annihilators.^75^ The effect can be attributed to slower diffusion of the annihilator itself because of the large and flexible crown ether moiety, in line with previous observations of the effect of alkyl substitutions on *k*_TTA_ *.* ^76^ Thus, the presence of the charge separated state does not affect the ability of the molecule to undergo upconversion.

Next, the dependence of the upconversion intensity, *I*_UC_, on the excitation intensity, *I*_ex_, was studied to ensure that subsequent experiments were performed in the regime where the upconversion quantum yield, Φ_UC_, is independent of fluctuations in *I*_ex_. This intensity range corresponds to a linear dependence of *I*_UC_ on *I*_ex_. It occurs above a threshold intensity, *I*_th_, defined as the point where half of the ^3^A* species are consumed through TTA,^77^ but below intensities where upconversion is saturated.^78^ The value of *I*_th_ was determined by fitting *I*_UC_ to *I*_ex_, using a model by Murakami and Kamada,^77^

$$I_{\mathrm{UC}}=a\left(1+\frac{1-\sqrt{1-8I_{\mathrm{ex}}/I_{\mathrm{th}}}}{4I_{\mathrm{ex}}/I_{\mathrm{th}}}\right)I_{\mathrm{ex}}$$ 8

where *a* is a dimensionless fitting parameter. From eq 8, the *I*_th_ value was determined to 0.88 W/cm^2^ in DCM (Figures 6d and S42). For reference, the intersection of low and high intensity power curves in a log–log plot occurs at 1.0 W/cm^2^. This traditional method correspond to half the value of *I*_th_ and a β of 0.38.^77^ To ensure linearity, subsequent experiments were done well above *I*_th_, but below the maximum *I*_ex_ of 6.7 W/cm^2^ probed here (≈ 5.3–6.2 W/cm^2^ with 60–70 mW excitation power and 1.2 mm beam diameter, Figure S43).

The quantum yield of upconversion, Φ_UC_, was calculated in accordance with the literature using an external reference (Figures S44 and S45). The sensitizer, PtOEP, in DCM and CHCl_3_, was used as the external reference (Φ_Ph_ = 0.415 in CHCl_3_),^64^ resulting in an Φ_UC_ of 0.0045 in DCM, and 0.0042 in a 1:1 DCM:methanol mixture. To examine how the CSS is affecting TTA, the Φ_TTA_ was calculated using eq 1. In DCM, Φ_F_ = 0.053, Φ_TET_ = 0.94, and Φ_exc_ = 0.60, resulting in an Φ_TTA_ of 0.15. This is in line with the expectation for a 9-substituted anthracene annihilator. Φ_TTA_ for typical anthracene and 9-and 10-substituted anthracene annihilators range between 0.02 and 0.24 at this concentration.^66,79,80^ The value of Φ_TTA_ is expected to be in the same range in the 1:1 DCM:methanol mixture based on a similar Φ_UC_, Φ_exp_ (0.58), viscosity and Φ_F_ (0.047 in methanol vs 0.053 in DCM). The significance of these results is that a CSS does not affect the ability of an annihilator to perform upconversion, it is only the emission quantum yield that is affected.

### TTA-UC Titrations

Having investigated the effect of a CSS on TTA-UC, we applied upconverted fluorescence to the sensing of K^+^ in DCM:methanol mixtures. The choice of solvent was motivated by a balance between sensitizer solubility, salt solubility, and the higher sensitivity and binding constant in the lower polarity solvent. Steady state upconversion spectra were taken in a solution of **1** and PtOEP (90 and 5 μM, respectively) with increasing proportions of KOAc (Figure 7a). The corresponding binding curves display the recorded signals using both prompt fluorescence and upconverted emission (Figure 7b). Notably, prompt fluorescence shows quantitative binding as expected at these annihilator concentrations, while the upconverted emission indicates much weaker binding, with *K* on the order of 10^4^ M^–1^. A likely explanation for the discrepancy is that K^+^ dissociates from the crown ether before annihilation occurs. The dissociation constant, *k*_d_, of K^+^ in 18-crown-6 ether has been determined to 10^2^–10^4^ s^–1^ in THF/polycarbonate, THF/methanol and pure methanol (with faster rates in more polar solvents).^81^ This is comparable to the triplet decay rate of **1,** ∼10^3^ s^–1^. Thus, it is plausible that the measured *K* reflects the equilibrium constant at the triplet surface of **1**, illustrating the danger of extrapolating physical data on different electronic surfaces.

**Figure 7:**
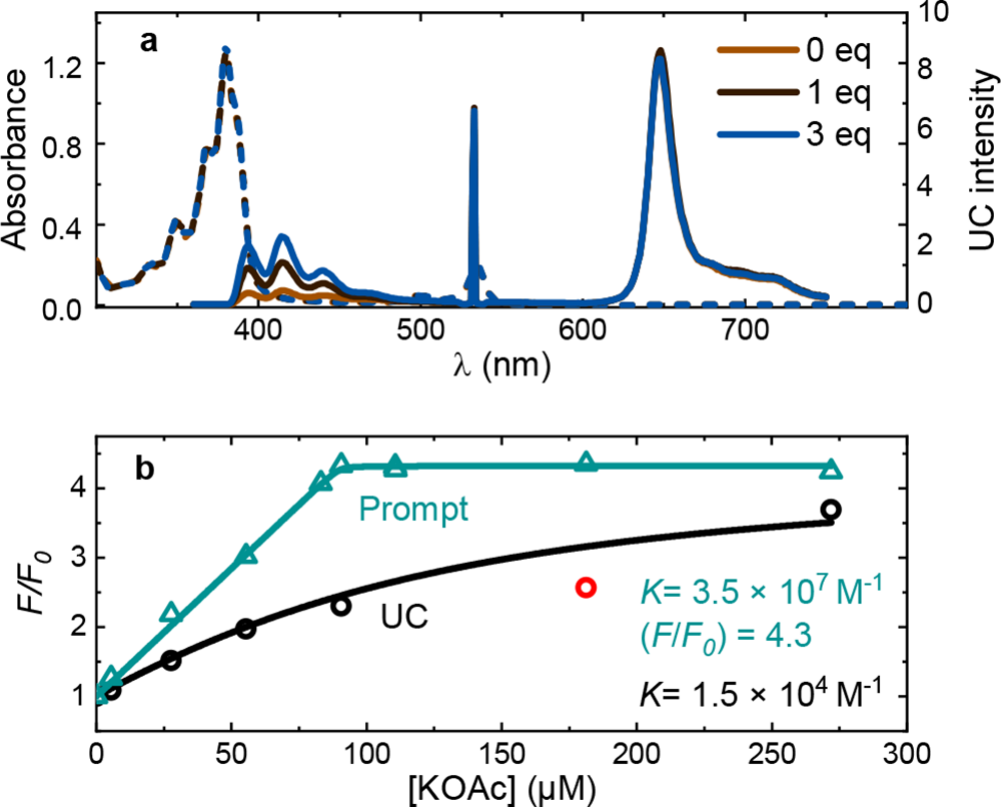
Titration data for **1** and PtOEP (90 and 5 μM). (a) Absorbance (dashed) and emission (solid) spectra with the sharp peak at 532 nm corresponding to the laser pulse. (b) TTA-UC and prompt *F*/*F*_0_ against KOAc concentration together with a fit to eq 2. The red data point was considered as an outlier.

The change in sensitivity implies that the practical dynamic range can be tuned by altering the sensitizer concentration. A higher sensitizer concentration will result in a quadratic increase in the rate of TTA (*k*_TTA_[^3^A*]^2^), thereby greatly limiting the chance of K^+^ dissociation and maintaining a sensitivity similar to that with prompt fluorescence. Conversely, a lower sensitizer concentration may be suitable when the ion concentration is large, since small deviations in the binding curve will give a smaller error in concentration.

### Sensing in a Microfluidic Device

A microfluidic device was made in order to evaluate how upconversion based sensing could practically be performed (Figure 8 and S46). A glass chip with three inlet channels was used. These were connected to three syringe pumps containing (1) a high concentration sensitizer and annihilator solution (40 and 360 μM, respectively), (2) pure solvent (1:1 DCM:methanol), (3) a KOAc solution (360 μM). By keeping the total injection rate constant (160 μL/min) and varying the flow rate of solvent and salt syringes, the [K^+^] could be seamlessly varied. Upconverted emission was coupled via a liquid light guide to a spectrofluorimeter, and the envelope of emission is anthracene-like (Figure 9a). Furthermore, the intensity of upconversion depends on [K^+^], and shows a similar binding constant to K^+^ as in the static upconversion experiments (Figures 9b and 7).

**Figure 8:**
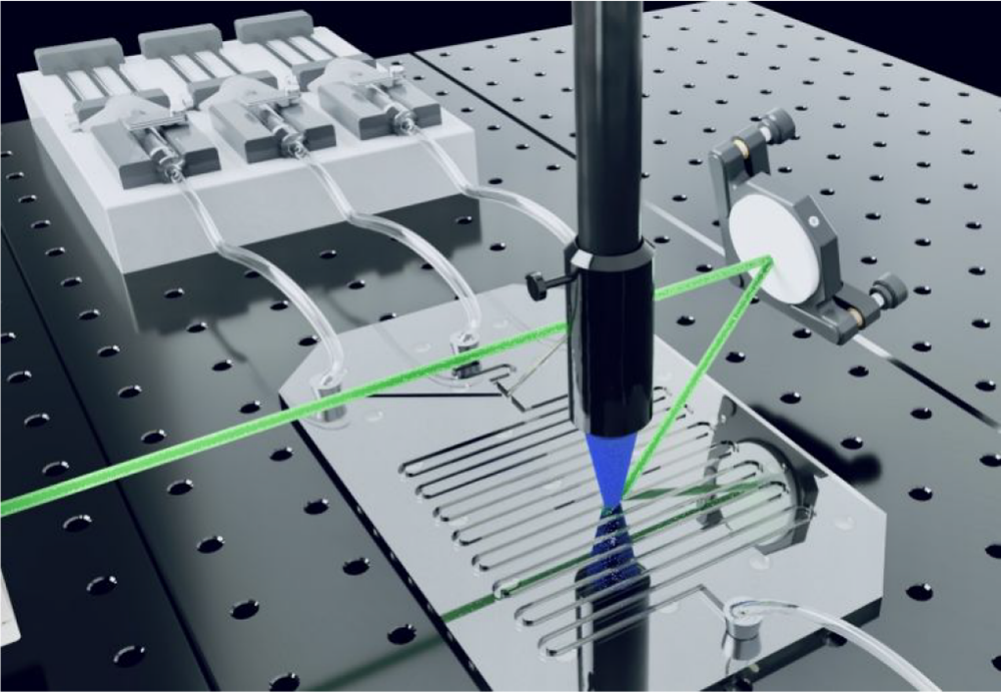
Illustration of the microfluidic setup, including microfluidic channel, syringes and tubing, laser beam, reflective mirror with kinematic mount, and lens attached to a liquid lightguide. The CAD file for the mirror and mount was obtained from Thorlabs, Inc. (KM200-E02 – Kinematic Mirror Mount for Ø2” Optics with Visible Laser Quality Mirror).^82,83^ For a photo of the full setup, see Figure S46.

**Figure 9:**
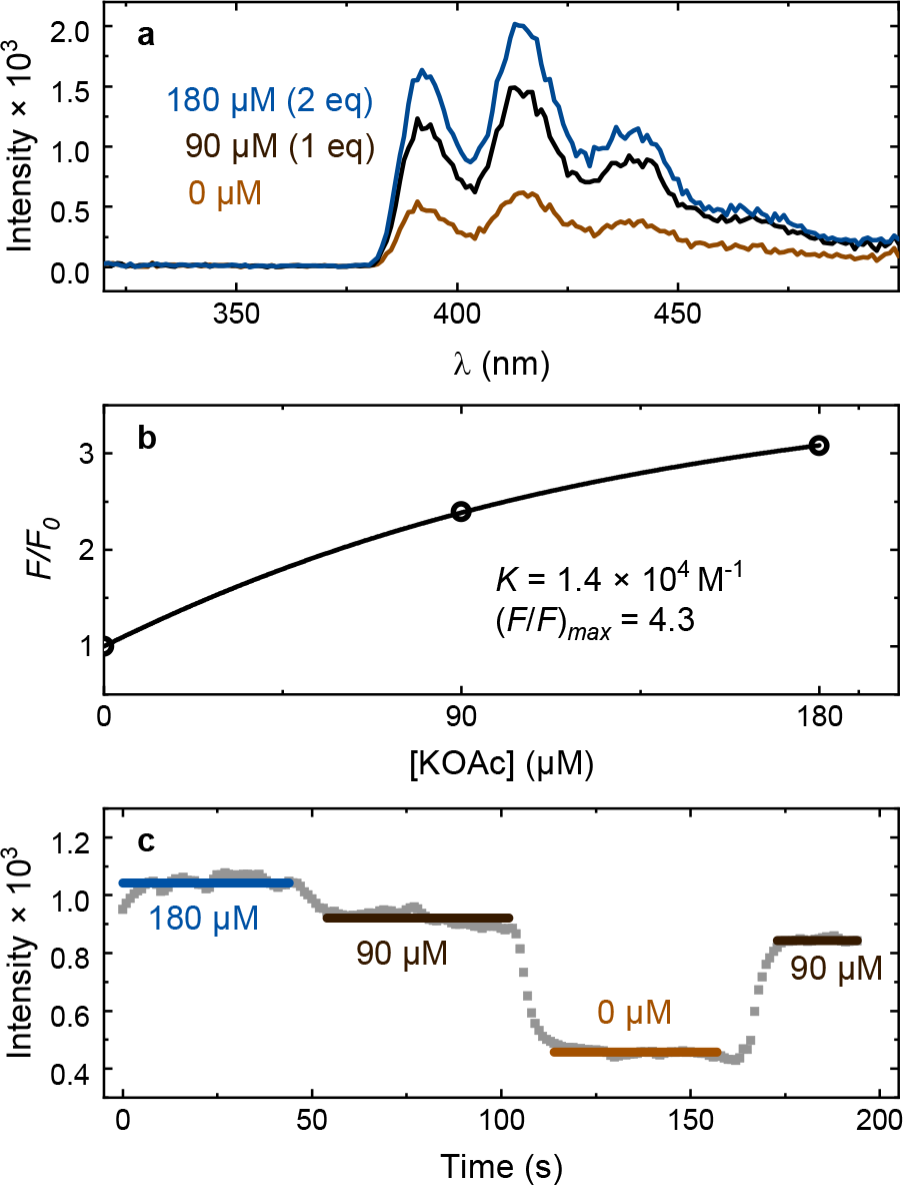
(a) Upconversion spectra and (b) binding curve for TTA-UC titration in a microfluidic device with 90 μM of **1** and 10 μM of PtOEP in a 1:1 DCM:methanol mixture. (c) Emission intensity at 415 nm in flow, with step changes in the KOAc concentration every 60 s.

To evaluate the stability of the upconverted signal, the emission at 415 nm (corresponding to the strongest vibronic band) was monitored continuously during step changes of the K^+^ concentration (Figure 9c). This allowed monitoring of the signal at 180, 90, and 0 μM KOAc with a repeat at 90 μM to examine the repeatability. Within this range of salt concentrations, the upconversion signal stabilizes in about 10 s after the change in concentration. There is a slight downward trend in the signal over time because of small amounts of oxygen leaking into the system. The reduction when repeating the measurement at the same concentration (90 μM) after 2 min is 9%. Considering the simple setup with no additional purging or oxygen barriers, this semiquantitative result is promising for future developments of microfluidic sensing. Oxygen may be limited further by applying additional enclosures and/or inlets for purging with an inert gas. Furthermore, by using upconverted rather than prompt emission we avoid most scattering from the glass surface, enabling artifact free spectra (Figure 9a). This allows the use of an external diode without installing waveguide optics or other costly customizations. With these initial results, we hope to further stimulate the development of upconversion-based sensing in microfluidics.

## Conclusions

Two anthracene-based PET sensors were tested in terms of sensitivity, dynamic range, and selectivity for K^+^. Upconverted emission was used as the binding indicator in order to enable long wavelength excitation and avoid scattering and autofluorescence in sensing devices. Experiments and TD-DFT show that a CSS quenches the anthracene fluorescence. However, with K^+^ complexation, the CSS increases in energy, making the molecule more emissive. The applicability of upconversion-based sensing was evaluated by comparing the kinetics and quantum yields of the main steps of TTA-UC in salt-free and K^+^-saturated solutions. The CSS was found not to affect any process in the upconversion pathway. It only affected the emission quantum yield, indicating that PET based sensors are compatible with an upconversion mechanism.

The sensitivity obtained when measuring upconverted emission was lower compared to prompt fluorescence. This unexpected result can be attributed to the fact that association and dissociation occurs on the same time scale as the triplet lifetime. Thus, the system has time to reach a new equilibrium on the triplet surface. Consequently, the sensitivity is dependent on how the system reaches the emissive state.

Lastly, we successfully applied upconversion in a microfluidic device, thereby reducing measurement time and improving the tuneability of analyte concentrations. For in vitro biosensing applications the method has the advantage that it avoids scattering and autofluorescence and relies on relatively cheap and lightweight equipment that can be modified based on specific needs.

## Supplementary Material



## Data Availability

The raw data from this study is stored at the Swedish National Data Service with the digital object identifier 10.5878/3nrz-tn49 (https://doi.org/10.5878/3nrz-tn49).

## Notes

Materials and methods; synthesis; mechanism of triplet-triplet annihilation; Rehm–Weller theory; supplementary figures and tables; and references (PDF)
