# Supplementary material for: Excitation-Dependent K+ Sensing by Combining Photoinduced Electron Transfer and Triplet–Triplet Annihilation
Source: J Phys Chem A. 2025 Jun 10;129(24):5326–36. doi: 10.1021/acs.jpca.5c02938 (PMC12186624; doi:10.1021/acs.jpca.5c02938)
Supplement: Supplementary file 1 [file jp5c02938_si_001.pdf]

Supporting Information for:

Excitation-Dependent K^+ Sensing by Combining Photoinduced Electron Transfer and Triplet-Triplet Annihilation

Hannah Tideland,[a] Andrew J. Carrod,[a] Yuanxin Liang[a] and Karl Börjesson[a] *

[a] University of Gothenburg, Department of Chemistry and Molecular Biology, Medicinaregatan 7B, 41390, Gothenburg, Sweden.

*Email: karl.borjesson@gu.se

Table of Contents

1. Materials and Methods 2
2. Synthesis 6
3. The mechanism of triplet-triplet annihilation 8
4. Rehm-Weller theory 9
5. Supplementary Figures 10
6. Supplementary Tables 35
7. References 36

1. Materials and Methods

Synthesis: Starting materials and solvents were purchased from Sigma-Aldrich, VWR, or Fisher Scientific, and were used without further purification unless otherwise stated. All reactions were carried out using Schlenk techniques in oven-dried glassware, unless specifically stated. Solvents used for moisture- and oxygen-sensitive reactions were dried using an Inert PureSolv solvent purification system, or over molecular sieves (3Å). The sieves were obtained from commercial sources and dried at 300 °C for 24 h immediately before use. ^{1}H nuclear magnetic resonance (NMR) spectra were recorded on a Bruker spectrometer at 600 MHz where ^{13}C NMR spectra were recorded at 150 MHz. Chemical shifts are given in parts per million using tetramethylsilane as an internal standard.

Sample preparation: All samples for photophysical measurements were prepared in 3 mL sealable cuvettes. For absorption and prompt fluorescence, fresh stock solutions of the molecular sensor (1 mM) and salts (3 mM in polar solvents, 0.5-1 mM in DCM) were prepared and added to the solvent with a micropipette. TTA-UC samples were prepared in an MBraun glove box with oxygen and water levels no higher than 1 ppm. Cuvettes were sealed with screw caps and PTFE septa. In TTA-UC titrations a saturated $NaClO_4$ solution in DCM was used and diluted with DCM in suitable proportions to achieve a binding curve.

UV-Vis absorption: UV-Vis absorption was measured on a PerkinElmer Lambda650 spectrophotometer using D_2 and tungsten lamps as light sources and a photomultiplier tube (PMT) detector. The step size was set to 1 nm in all measurements. The solvent of each sample was taken as the background and subtracted. The same cuvette was used for solvent baseline and sample. Absorption spectra were taken for each salt concentration at the start and end of fluorescence titrations. Dilution was corrected for by dividing the absorption intensity with the ratio of current to initial molecular sensor concentration $[\text{sensor}]/[\text{sensor}]_0$.

Prompt fluorescence: Prompt fluorescence emission spectra were recorded on an Edinburgh FLS1000 spectrofluorometer, using a 450 W Xe lamp light source and a PMT detector. Samples were excited at 350 nm. The dwell time was set to 1.00 ns for quantum yield measurements and 0.20 ns for other spectra.

Stern-Volmer analysis: Phosphorescence quenching of the sensitizer (PtOEP) by the annihilator (molecules **1** and **2**) were measured on an Edinburgh FLS1000 spectrofluorometer, using a 450 W Xe lamp light source and a PMT detector. Samples were excited at 532 nm and the dwell time was set to 0.20 ns. The relative intensity of the maximum sensitizer emission (at 647 nm), I/I_0, was plotted against the annihilator (quencher) concentration and fit to the Stern-Volmer equation.

$$\frac{I_0}{I} = 1 + k_{TET}\tau_0[\text{A}], \qquad \text{(S1)}$$

where I_0 is the intensity maximum without annihilator, k_{TET} is the rate of triplet energy transfer (TET) and τ_0 is the lifetime of the sensitizer triplet in the absence of the annihilator.

The quantum yield of TET as a function of annihilator concentration was determined and fitted to the following relation.

$$\Phi_{TET} = \frac{k_{TET}[\text{A}]}{k_{TET}[\text{A}]+k_0} = 1 - \frac{I}{I_0} \qquad \text{(S2)}$$

where k_0 is the rate of triplet decay in absence of the annihilator.

Steady-state upconversion: Excitation power dependent TTA-UC spectra of molecule **1** (the annihilator) were recorded at powers from 3 to 76 mW on an FLS1000 spectrofluorometer, excited by a coherent laser from OBIS with a 532 nm excitation wavelength. Emission was collected 90 degrees from the excitation pathway. A 533 nm notch filter was used to protect the detector. The beam diameter was determined to 1.2 mm using an Ophir SP 932U beam profiling camera (Fig. S13). Two ND 2.0 filters were attached on the camera aperture to prevent damage. The diameter was set to four standard derivations from the center of the Gaussian profile, according to ISO guidelines.[1]

The threshold intensity was measured from the integrated upconversion emission spectra in the 320-500 nm region at excitation powers of 3 to 76 mW with an OBIS 532 nm cw laser diode and a PMT detector. The laser had a beam diameter of 1.2 mm.

Quantum yields: The emission quantum yields of the samples were measured according to

$$\Phi_s = \Phi_r \frac{(1-10^{-A_r})n_s^2 F_s}{(1-10^{-A_s})n_r^2 F_r}, \quad \text{(S3)}$$

where Φ_r is the known fluorescence quantum yield of the reference, A_i is the absorbance of sample ($i=s$) and reference ($i=r$), respectively, n_i is the refractive index, and F_i is the fluorescence intensity integrated over the wavelength range. The excitation source, wavelength and detector were the same for the reference and sample, hence the flux of excitation does not need to be considered in this calculation. 9,10-diphenylanthracene in cyclohexane (Φ_F = 0.97) was used as the standard for the fluorescence quantum yield measurement.[2] The excitation wavelength was set to 350 nm. Platinum(II) octaethyl porphyrin (PtOEP) in DCM (Φ_{Ph} = 0.415) was used as the standard for the upconversion quantum yield.[3] The excitation wavelength was set to 532 nm.

Time-resolved emission: Phosphorescence decays of the sensitizer was measured on an Edinburgh FLS1000 spectrofluorometer using a microsecond flash lamp as the excitation source with a 100 Hz repetition rate. The flash lamp had a pulse width of 1 – 2 μs and the response width of the PMT detector was 600 ps.

Upconverted fluorescence and sensitizer phosphorescence of sensitizer-annihilator solutions was measured on an Edinburgh FLS1000 spectrofluorometer with a q-switched Spectra- Physics Nd:YAG laser (INDI, 532 nm, pulse width ≈7 ns) coupled to a Spectra-Physics primoscan optical parametric oscillator (OPO) as the excitation source. The signal was detected on a PMT in MCS mode (using the FLS1000).

Transient absorption: Transient absorption spectra were recorded on an Edinburgh LP980 spectrometer with a SpectraPhysics Nd:YAG laser (INDI, 532 nm, pulse width ≈7 ns) coupled to a Spectra-Physics PrimoScan optical parametric oscillator (OPO) as the pump excitation source and a 150 W Xe arc lamp in pulsed mode (10 Hz, 6.0 ms pulse width) as the probe. An image intensified CCD camera (iCCD) was used as the detector for spectra and a PMT detector (Hamamatsu R928) was used for transient kinetics. The transient absorption spectra and time-resolved absorption of the sensitizer-annihilator mixture (ca 90 and 5 μM, respectively) in salt-free and K^+-salt saturated solutions were taken to estimate the initial concentration of annihilator triplets, [$^3A^*$], as described in the main text. The excited state absorption maximum of the sensitizer at 427-430 nm with 9 μs delay was found to provide the highest sensitizer signal. Due to a much higher signal to noise, the emission decay traces (Figures S17-S33) were used to obtain parameters τ_{TA} and β, rather than the excited state absorption.

Upconversion in microfluidic device: Upconversion titrations at constant flow rate were performed in a serpentine channel microfluidic chip from Micronit (140 × 250 μm, 15.2 μL total volume). Three separate syringes (Beckton-Dickinson, 5 mL, LUER-LOK™ , plastic) were used for injection of 1) upconversion solution of sensitizer (40 μM) and annihilator (360 μM), 2) solvent (1:1 DCM:methanol), and 3) KOAc (360 μM) solution in 1:1 DCM:methanol. A syringe was also connected to the outlet of the microfluidic channel for withdrawal of waste. The contents of the syringes were injected or withdrawn with syringe pumps from KD Scientific (OEM110). The total injection and withdrawal rate was 160 μL/min, with equal injection and withdrawal rates ensuring even pressure across the channel. The flow of the KOAc solution was varied between 0, 40 and 80 μL/min. Titrations were performed by recording the photon counts at the 0-1 band (415 nm) at different salt concentrations. The flow rate was changed every 60 seconds and the upconversion spectra were recorded after the signal at 415 nm had stabilized.

Derivation of the binding constant for host-guest equilibrium: Denoting the crown ether sensor molecule as H (host), the cation as G (guest), and the host-guest complex as HG, with concentrations in square brackets, the host-guest equilibrium can be written as:

$$\mathrm{H} + \mathrm{G} \underset{k_d}{\overset{k_f}{\leftrightharpoons}} \mathrm{HG}. \quad \text{(S4)}$$

The equilibrium constant for the host-guest association (i.e., the binding constant) is defined as:

$$K = \frac{[\mathrm{HG}]}{[\mathrm{H}][\mathrm{G}]}. \quad \text{(S5)}$$

The total concentration of host, $[\mathrm{H}]_0$ and guest, $[\mathrm{G}]_0$ follow the relationships,

$$[\mathrm{H}]_0 = [\mathrm{H}] + [\mathrm{HG}] \quad \text{(S6)}$$

$$[\mathrm{G}]_0 = [\mathrm{G}] + [\mathrm{HG}] \quad \text{(S7)}$$

Inserting Equation S6 and S7 into Equation S5 gives a quadratic expression for [HG]:

$$K = \frac{[\mathrm{HG}]}{([\mathrm{H}]_0 - [\mathrm{HG}])([\mathrm{G}]_0 - [\mathrm{HG}])} \Leftrightarrow [\mathrm{HG}]^2 - ([\mathrm{H}]_0 + [\mathrm{G}]_0 + 1/K)[\mathrm{HG}] + [\mathrm{H}]_0[\mathrm{G}]_0 = 0. \quad \text{(S8)}$$

This has the following solutions:

$$[\mathrm{HG}] = \frac{1}{2}([\mathrm{H}]_0 + [\mathrm{G}]_0 + 1/K) \pm \sqrt{\frac{1}{4}\left([\mathrm{H}]_0 + [\mathrm{G}]_0 + \frac{1}{K}\right)^2 - [\mathrm{H}]_0[\mathrm{G}]_0}\,. \quad \text{(S9)}$$

For physical concentrations ($[\mathrm{H}]_0 \geq 0$, $[\mathrm{G}]_0 \geq 0$), only the negative sign gives a valid solution. (The positive solution gives a nonzero value when $[\mathrm{G}]_0 = 0$.)

The total fluorescence is a sum of the emission from all fluorescent compounds, i.e., in this case where the host and host-guest complex are fluorescent (but not the guest):

$$F = k_H[\mathrm{H}] + k_{HG}[\mathrm{HG}]. \quad \text{(S10)}$$

The fluorescence in the absence of the guest is:

$$F_0 = k_H^0[\mathrm{H}]_0. \quad \text{(S11)}$$

Combining Equation S6, with Equations S10 and S11, the relative fluorescence is:

$$\frac{F}{F_0} = \frac{k_H[\mathrm{H}] + k_{HG}[\mathrm{HG}]}{k_H^0[\mathrm{H}]_0} = \frac{1}{[\mathrm{H}]_0}\left(\frac{k_H}{k_H^0}([\mathrm{H}]_0 - [\mathrm{HG}]) + \frac{k_{HG}}{k_H^0}[\mathrm{HG}]\right) = \frac{k_H}{k_H^0} + \frac{[\mathrm{HG}]}{[\mathrm{H}]_0}\left(\frac{k_{HG}}{k_H^0} - \frac{k_H}{k_H^0}\right). \quad \text{(S12)}$$

Further, inserting the expression for [HG] in (Equation S9) gives:

$$\frac{F}{F_0} = \frac{k_H}{k_H^0} + \frac{0.5([\mathrm{H}]_0 + [\mathrm{G}]_0 + 1/K) - \sqrt{0.25\left([\mathrm{H}]_0 + [\mathrm{G}]_0 + \frac{1}{K}\right)^2 - [\mathrm{H}]_0[\mathrm{G}]_0}}{[\mathrm{H}]_0}\left(\frac{k_{HG}}{k_H^0} - \frac{k_H}{k_H^0}\right). \quad \text{(S13)}$$

Equation S13 was used to fit F/F_0 to $[\mathrm{G}]_0$, i.e., the added cation (guest) concentration. Note that $[\mathrm{HG}]/[\mathrm{H}]_0$ approaches unity at very high $[\mathrm{G}]_0$ and binding constants, K. Thus, k_{HG}/k_H corresponds to the asymptotic maximum value of F/F_0. This is denoted $(F/F_0)_{\max}$ in the main text.

Estimation of kinetic upconversion parameters: The triplet lifetime of the annihilator, and the rate of TTA was determined by modelling the triplet decay to the differential equation:

$$\frac{d[^3\mathrm{A}^*]}{dt} = -k_{TA}[^3\mathrm{A}^*] - k_{TTA}[^3\mathrm{A}^*]^2 \quad \text{(S14)}$$

where $k_{TA} = 1/\tau_{TA}$ is the rate of triplet decay from all first-order decay sources, and k_{TTA} is the rate of TTA. This gives the time-dependent emission intensity mentioned in the main text (Equations. 6 and 7):

$$I(t) \propto [^3\mathrm{A}^*]^2 = \left([^3\mathrm{A}^*]_0 \frac{1-\beta}{\exp(t/\tau_{TA}) - \beta}\right)^2, \quad \text{(S15)}$$

where,

$$\beta = \frac{2k_{TTA}[^3\mathrm{A}^*]_0}{1/\tau_{TA} + 2k_{TTA}[^3\mathrm{A}^*]_0}. \quad \text{(S16)}$$

Here, $[^3\mathrm{A}^*]_0$is the triplet annihilator concentration at $t = 0$. This parameter was determined from the transient absorption maximum at 430 nm at 15 µs delay (9.36×10^{-7} M, Figure 6d) and the absorption coefficient of the anthracene triplet in cyclohexane. The value was taken at a delay corresponding to the decay of the sensitizer excited state absorption due to TET.

Density functional theory (DFT) calculations: Calculations with time-dependent density functional theory (TD-DFT) were done in the Gaussian 16 package to investigate the presence of charge separated states in the singlet and triplet surfaces and estimate their energies and transition probabilities.[4-8] 5 excited states each of singlet and triplet character were calculated to improve the stability of the excited state energies and identify the charge-separated state (CSS) and locally excited state (LES) on the singlet surface. The configuration interaction (CI) coefficients of the relaxed state show the contribution of prominent transitions to the excited state. The square of the CI coefficient represents the probability of the transition. Only CI coefficients above 0.1 (>1 % transition probability) are listed here (Table S1). Note that the HOMO-LUMO transition corresponds to the n-π^* charge-separated state (CSS) in the free molecule. Conversely, the HOMO-LUMO transition is the anthracene-localized π-π^* transition in the sensor-ion complex. Finally, it should be noted that the relaxed CSS of 1-Na^+, 2-K^+, and 2-Na^+ could not be obtained, since calculations did not converge to the same state. During iterations of the geometry optimization the CSS changed order with other higher excited states, even when using tight convergence criteria. This is typical where there are avoided crossings or conical intersections on the potential energy surface.[9] Confirmation of such features requires higher level of theory such as CASSCF which is beyond the scope of this study. However, the failure of TD-DFT calculations to generate a stable minimum on the CSS surface is indicative of a much lower stability of the state in the ion-complexing molecules.

2. Synthesis

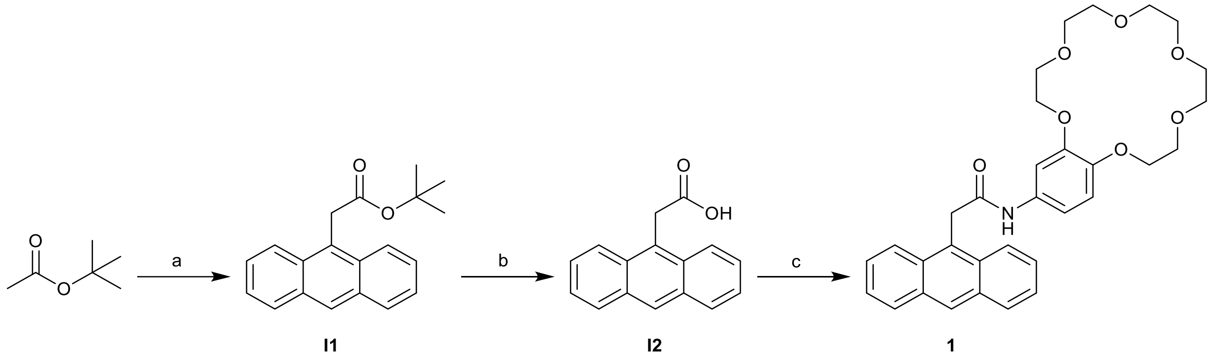

Scheme S1: Synthetic route to anthracene-based annihilator 1, where a) (i) LiHMDS, THF, 1 h, -78 °C, (ii) $ZnCl_2$, THF, 0.5 h, -78 °C (iii) $Pd(dba)_2$, $P(t\text{-}Bu)_3$, 9-bromoanthracene, 12 h, 80 °C; b) TFA, DCM, RT, 4 h; c) RNH_2, DCC, DMF, 0 °C → RT, 18 h.

I1

The compound **I1** was synthesised using a method modified from that reported by Grabowski et al.[10] A flask was charged with LiHMDS (14 mL, 1 M in THF), and cooled to -78 °C. Tert-butylacetate (1.91 mL, 14 mmol) was added dropwise, and the mixture stirred at -78 °C for a further 1 hour. To the solution containing lithium enolate was added dropwise a solution of $ZnCl_2$ (3.83 g, 28.1 mmol) in THF (10 mL), stirring was continued for 30 mins at -78 °C. After which time, $Pd(dba)_2$ (100 mg, 0.17 mmol), 9-bromoanthracene (3 g, 11.67 mmol), and $P(t\text{-}Bu)_3$ (200 mg, 0.98 mmol) were added dropwise as a solution in THF (10 mL). The reaction was allowed to warm to room temperature, before heating to 80 °C. The reaction was allowed to maintain this temperature with stirring for 12 hours. After cooling, water was added (100 mL) and then the mixture was extracted with Et_2O (3 x 50 mL). The combined organic layers were dried, and the solvent removed under reduced pressure. The crude product was subjected to column chromatography and combined fractions were dried to obtain a pale yellow solid. (1.8 g, 6.2 mmol, 54 %) ^1H NMR ($CDCl_3$, 600 MHz): δ (ppm) = 8.42 (m, 1H), 8.31 (d, 2H, J = 8.8 Hz), 8.01 (d, 2H, J = 8.8 Hz), 7.54 (td, 2H J = 7.7 Hz, 1.1 Hz), 7.47 (td, 2H, J = 7.8 Hz, 1.1 Hz), 4.54 (s, 2H), 1.38 (s, 9H).

I2

Compound **I1** (1.0 g, 3.4 mmol) was dissolved in DCM (20 mL) in a N_2-purged flask. Trifluoroacetic acid (TFA, 0.6 mL, 7.8 mmol) was added to the solution and stirred at room temperature for 6.5 hours. Then 20 % NaOH (10 mL) was added to the reaction mixture and the aqueous layer was washed with DCM twice. Combined organic layers were washed with NaOH (20 %) three times. To the combined aqueous layers was added concentrated H_2SO_4, resulting in the precipitation of a pale yellow solid. The solid was filtered off, washed with water and dried under vacuum overnight yielding a pale-yellow dry powder (485 mg, 2.1 mmol, 62 %). ^1H NMR ($CDCl_3$, 600 MHz) *δ* (ppm) = 8.43 (s,1H), 8.21 (d, 2H, *J* = 8.6 Hz), 8.01 (d, 2H. *J* = 8.1 Hz), 7.53 (t, 2H, *J* = 7.2 Hz), 7.46 (t, 2H, *J* = 7.0 Hz), 4.63 (s, 1H).

1

4′-Aminobenzo-18-crown-6 (150 mg, 0.46 mmol), **I2** (90 mg, 0.38 mmol), and *N,N′*-dicyclohexylcarbodiimide (DCC, 100 mg, 0.48 mmol) were dissolved in DMF (5 mL), and stirred at 0 °C for 1 hour, before slowly warming to room temperature and stirring for a further 18 hours. The solvent was then evaporated, and the resulting solid recrystallized from a methanol:pentane mix (1:2). The precipitate was collected and further washed with further ice cold methanol:pentane, to yield an off white solid (25 mg, 0.05 mmol, 13 %) ^1H NMR ($CDCl_3$, 600 MHz) *δ* (ppm) = 8.53 (s, 1H), 8.27 (d, 2H, *J* = 8.8 Hz), 8.10 (d, 2H, *J* = 8.4 Hz), 7.61 (t, 2H, *J* = 7.7 Hz), 7.54 (t, 2H, *J* = 7.4 Hz), 7.02 (d, 1H, *J* = 2.5 Hz), 6.76 (s, 1H), 6.65 (d, 1H, 8.8 Hz), 6.48 (dd, 1H, *J* = 8.5 Hz), 4.73 (s, 2H), 4.08-4.00 (m, 4H), 3.84 (t, 4H, *J* = 4.6 Hz), 3.74-3.61 (m, 12 H). ^{13}C NMR ($CDCl_3$, 151 MHz) 168.8, 149.0, 131.6, 131.5, 130.7, 129.5, 128.3, 127.2, 125.4, 123.7, 114.5, 112.7, 107.4, 70.7, 69.6, 69.5, 69.0, 37.5, 29.7. MS (ESI^+) found: 546.249 g/mol, predicted neutral species $C_{32}H_{35}NO_7$ 545.24135 g/mol.

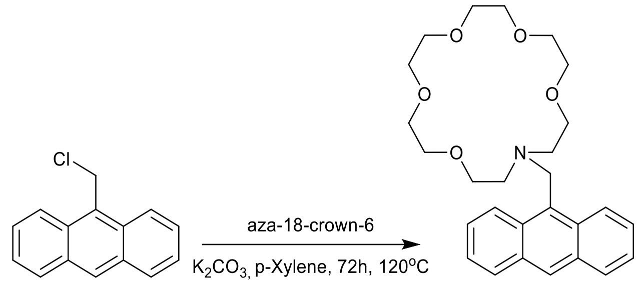

2

Scheme S2: Synthetic route to anthracene-based annihilator 2

2

The method was adapted from Bissell *et al.*[11] Briefly, 9-chloromethylanthracene (52.7 mg, 0.234 mmol) was dissolved in dry, hot p-xylene (3 mL). Aza-18-crown-6 (63.0 mg, 0.239 mmol) and potassium carbonate (35.9 mg, 0.258 mmol) were added, followed by stirring at 120°C for 72 h. The hot solution was filtered. After cooling, the filtrate was extracted with 4 M hydrochloric acid three times. The acid extract was neutralized with potassium carbonate and extracted with chloroform twice. The chloroform extract was dried over sodium sulfate and evaporated. The yellow-brown solid product was dried overnight under vacuum (30.1 mg, 0.066 mol, 28.3%). Spectroscopic data matches that previously reported.[11] ^{1}H NMR ($CDCl_3$, 600 MHz): δ (ppm) = 8.57 (d, 2H, J = 8.8 Hz), 8.40 (s, 1H), 7.98 (d, 2H, J = 8.3 Hz), 7.50 (t, 2H, J = 7.6 Hz), 7.45 (t, 2H, J = 7.6 Hz), 4.62 (s, 2H), 3.73-3.55 (m, 24 H), 2.90 (t, 4H, J = 5.8 Hz). MS (ESI$^+$) found: 454.2584 g/mol, predicted neutral species $C_{27}H_{35}NO_5$ 453,251524 g/mol.

3. The mechanism of triplet-triplet annihilation

TTA-UC is a photophysical process that through a series of steps produces a higher energy emission than that of the absorption (Figure 1). The process requires a sensitizer, S, and an annihilator, A. The sensitizer first absorbs a photon and is promoted to an excited singlet state, $^1S^*$. It then rapidly undergoes intersystem crossing (ISC) to a lower triplet state, $^3S^*$. When $^3S^*$ is close enough for orbital overlap (e.g., through diffusion) with ^1A, the triplet energy can be transferred through a Dexter mechanism, known as triplet energy transfer (TET). Two $^3A^*$ in proximity can form a pair that then undergoes a second Dexter energy transfer event. This results in one species being promoted to a higher excited state while the other returns to the ground state. This step is called triplet-triplet annihilation (TTA). Spin statistics dictate that the higher excited species will be either a singlet, a higher triplet or a quintet in ratios 1:3:5. Thus., only 1/9 of TTA events result in a fluorescent state. However, the triplet pair state with quintet character tends to dissociate rather than form a quintet monomer from TTA of two equal molecules.[12 13] Conversely, the second triplet state, $^3A^*_2$, tends to be reachable and can undergo effective internal conversion back to $^3A^*$, resulting in a theoretical singlet fraction of 2/5.[13]

The quantum yield of the upconversion process is the product of the quantum yields of the intermediate photophysical processes.

$$\Phi_{UC} = \Phi_F \Phi_{TET} \Phi_{TTA} \Phi_{exp} \tag{S17}$$

where Φ_F, Φ_{TET}, and Φ_{TTA} are the quantum yields of fluorescence, triplet energy transfer, and triplet-triplet annihilation, respectively. The last factor, Φ_{exp}, signifies the outcoupling losses such as the inner filter effect and scattering.[14] Note that the triplet-triplet annihilation quantum yield, Φ_{TTA}, has a maximum value of 0.5. This factor includes the fraction of triplet pairs that form the excited singlet state, known as the spin-statistical factor. An essential factor to ensure quantitative upconversion measurements is the threshold intensity, I_{th}. This is the excitation intensity, I_{ex}, at which the upconversion intensity, I_{UC}, goes from a quadratic dependence on I_{ex} to a linear dependence. It corresponds to the transition from when the rate of decay is dominated by non-radiative processes to when second order process TTA dominates. Importantly, above I_{th}, Φ_{UC} becomes independent on I_{ex}, [15] enabling direct quantitative comparison of upconversion yields.

4. Rehm-Weller theory

The trends in the dynamic range can be rationalized with the effect of the solvent polarity on the CSS energy. The free energy of the CSS can be expressed as: [16-18]

$$\Delta G_{CS} = IP - EA - \frac{e^2}{8\pi\epsilon_0}\left(\frac{1}{R_D} + \frac{1}{R_A}\right)\left(1 - \frac{1}{\varepsilon}\right) + \frac{1}{4\pi\varepsilon_0\varepsilon d}. \quad \text{(S18)}$$

where IP is the ionization potential of the donor, EA is the electron affinity of the acceptor, e is the elementary charge, R_D and R_A are the radii of donor (the nitrogen atom) and acceptor (anthracene), respectively, ε is the relative permittivity of the solvent, ε_0 is the vacuum permittivity, and d is the center-to-center distance between donor and acceptor. The ionization potential increases when a cation binds to the crown ether cavity. Thus, Equation S18 predicts a higher energy of the CSS in the ion complex than for the unbound state, in qualitative agreement with our experimental and computational results. This indicates that the low fluorescence quantum yield in the unbound states is due to PET, which is hindered by ion complexation. Finally, we note that the binding can be assumed to be static because PET and fluorescence occurs at much faster timescales than the diffusion length at these concentrations.

5. Supplementary Figures

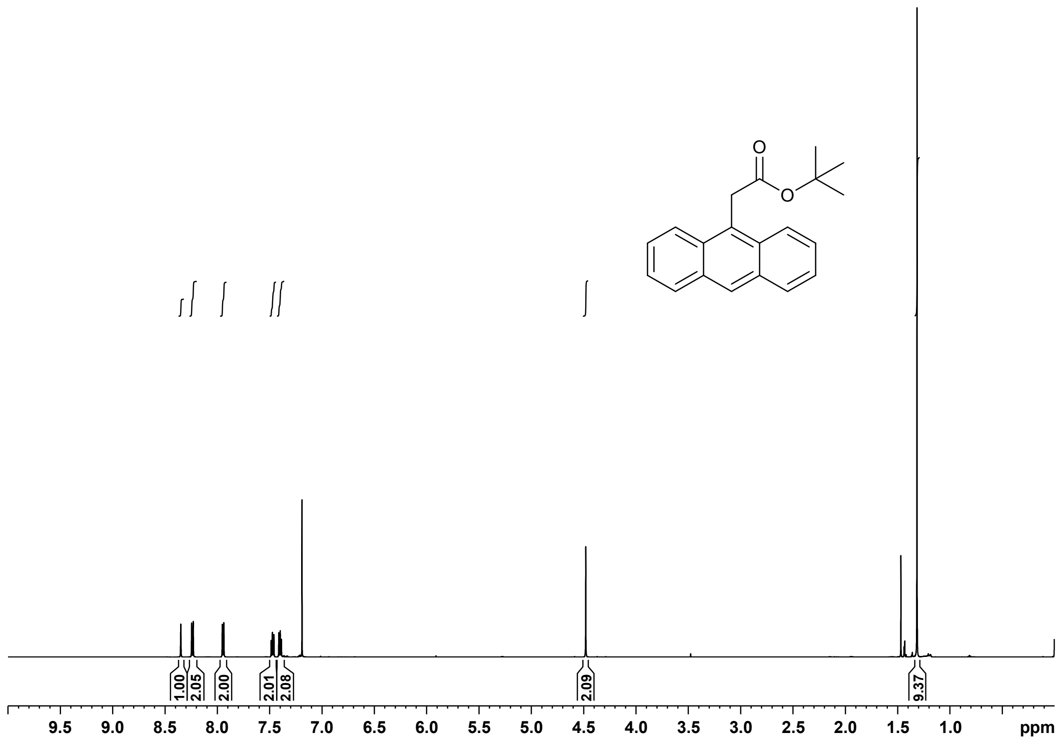

Figure S1: ^{1}H NMR spectrum for compound I1 taken at 600 MHz in $CDCl_3$.

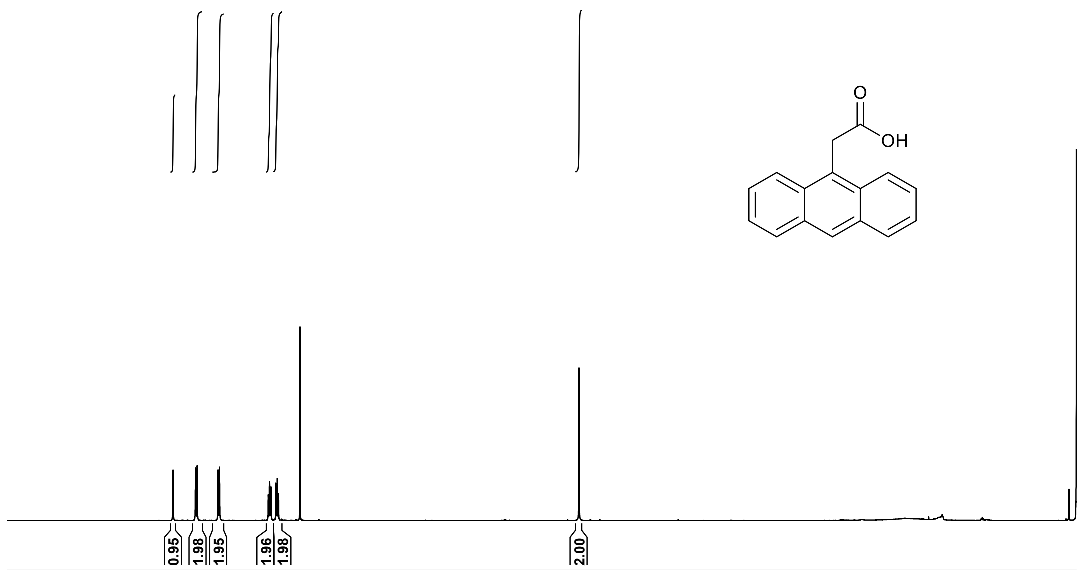

Figure S2: ^{1}H NMR spectrum for compound I2 taken at 600 MHz in $CDCl_3$.

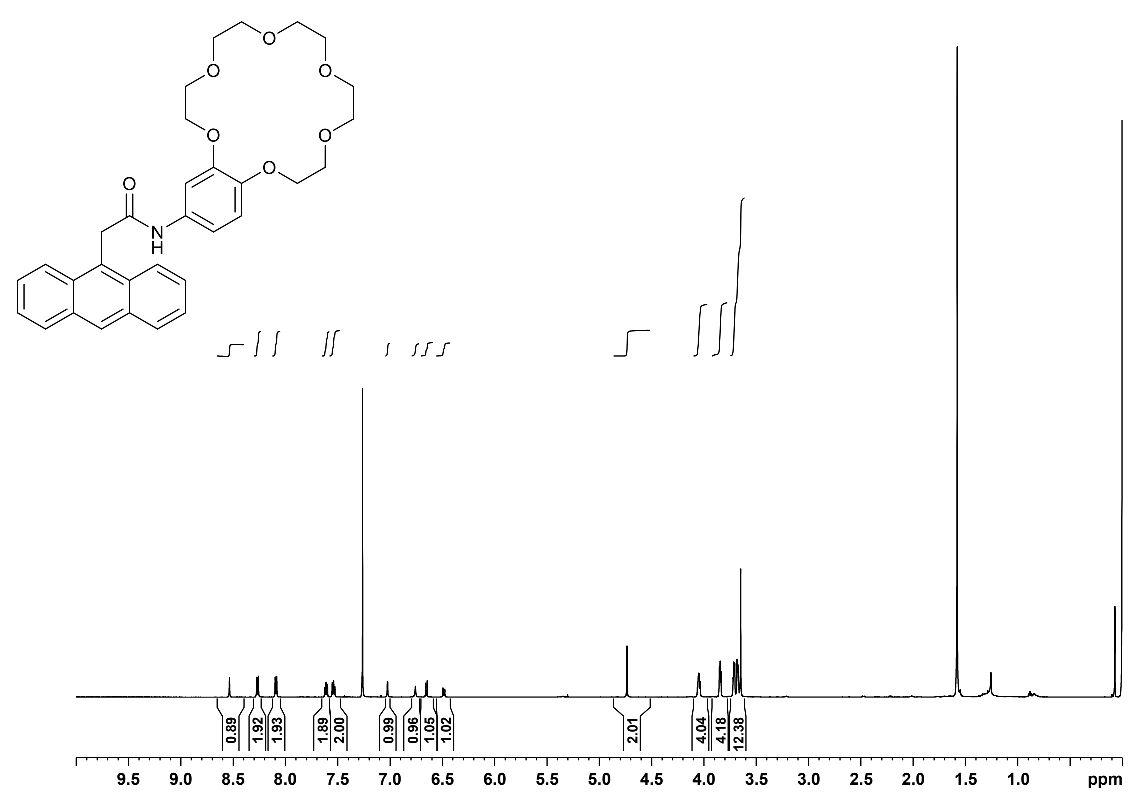

Figure S3: ^{1}H NMR spectrum for compound 1 taken at 600 MHz in $CDCl_3$.

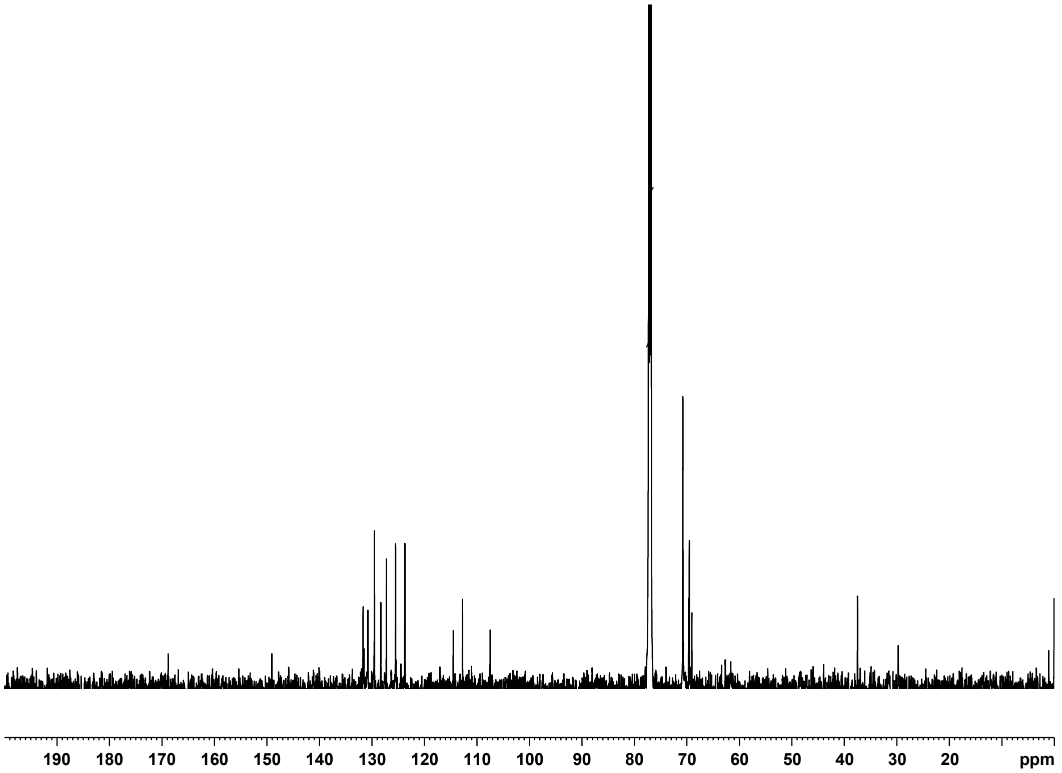

Figure S4: ^{13}C NMR spectrum for compound 1 taken at 151 MHz in $CDCl_3$.

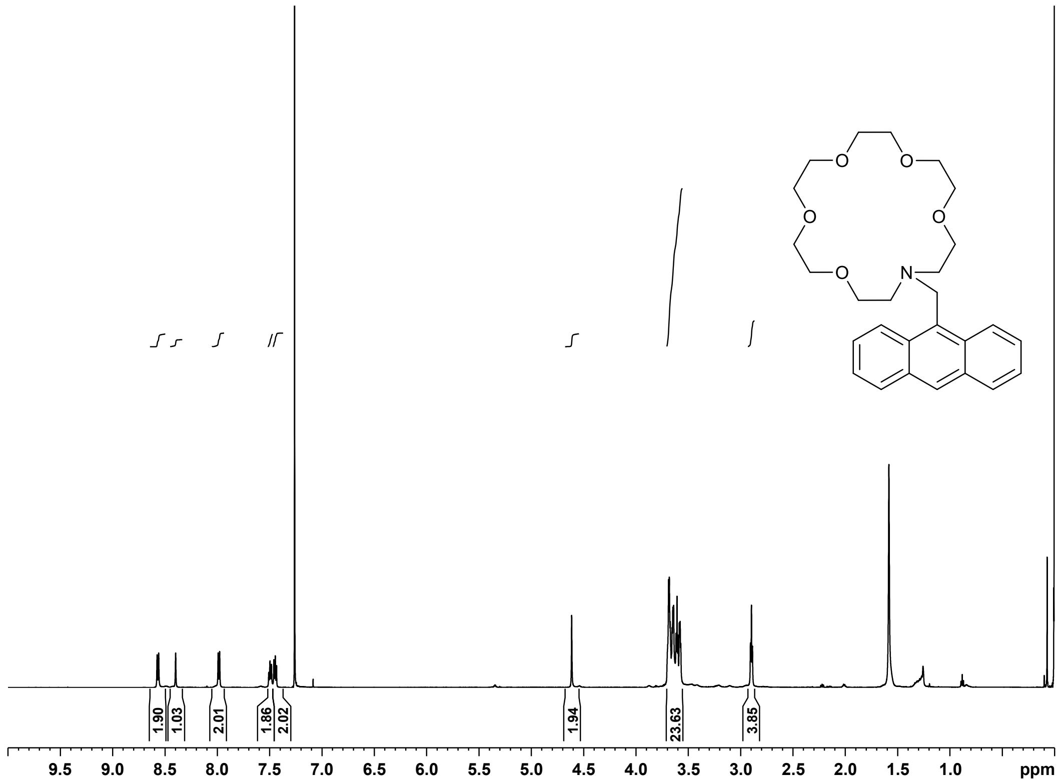

Figure S5: ^{1}H NMR spectrum for compound 2 taken at 600 MHz in $CDCl_3$.

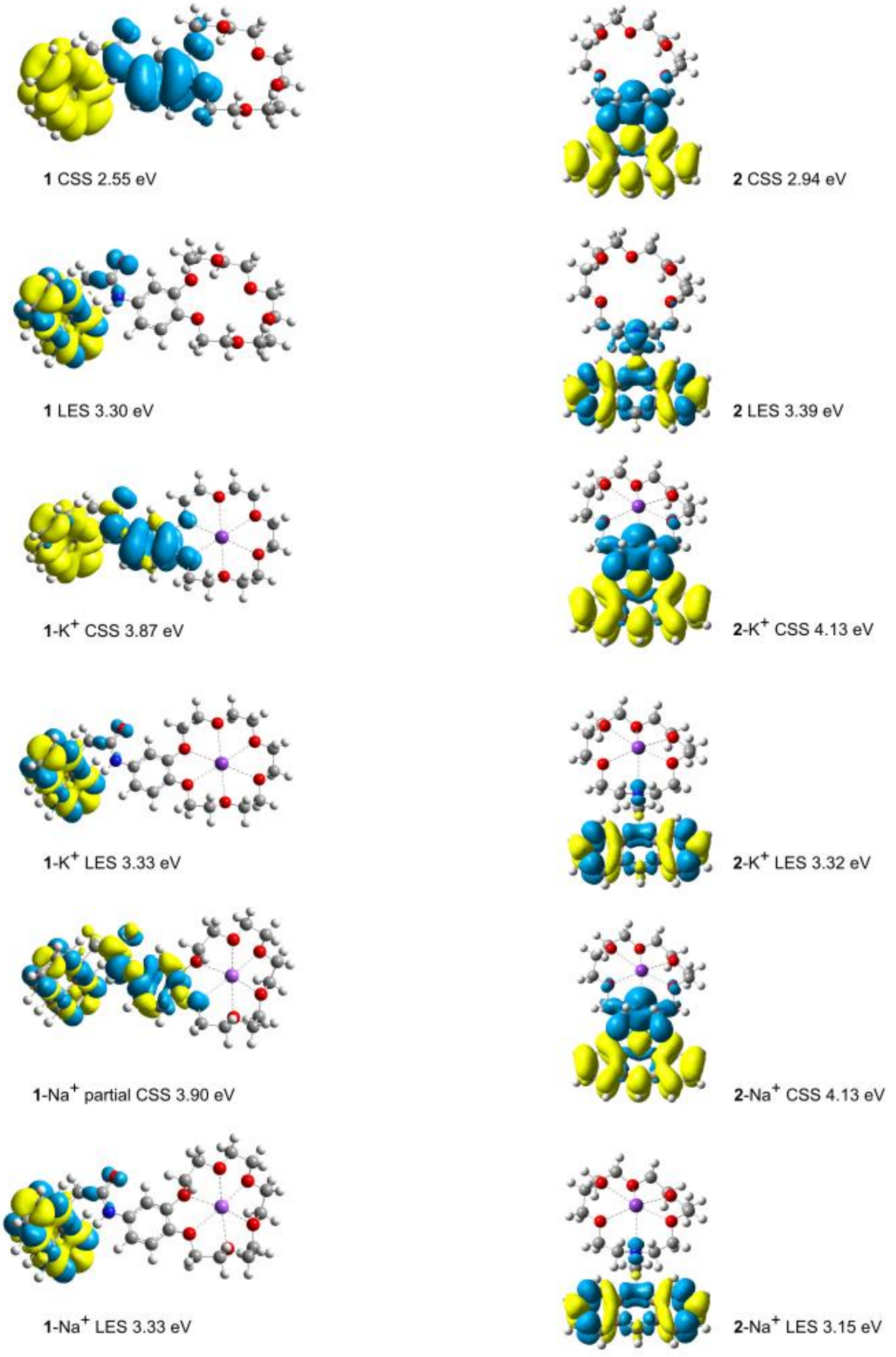

Figure S6: Electron density difference maps of the vertical excited singlet states corresponding to the lowest bright locally excited state (LES) and dark CSS of molecule **1** and **2** in the free, and K^+- and Na^+ complexing forms.

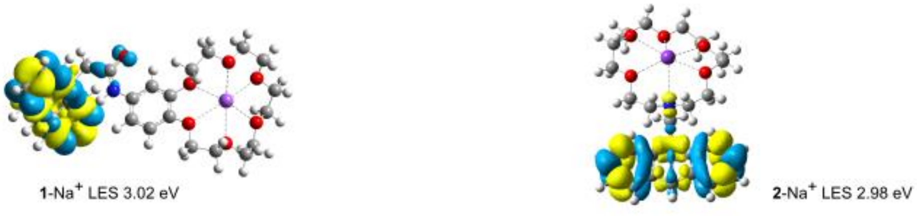

Figure S7: Electron density difference maps of the relaxed singlet excited states corresponding to the lowest bright locally excited state (LES) of molecule **1** and **2** in the Na^+ complexing forms.

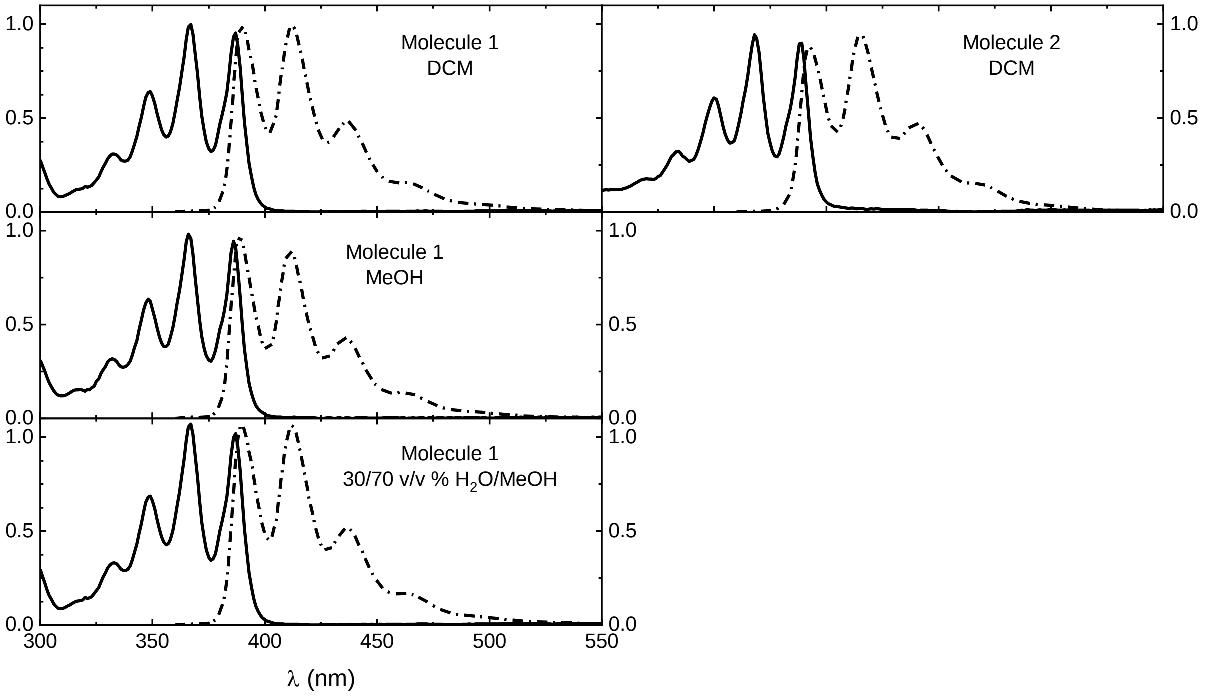

Figure S8: Molar extinction coefficient and emission spectra of molecules **1** and **2** in the different solvents used.

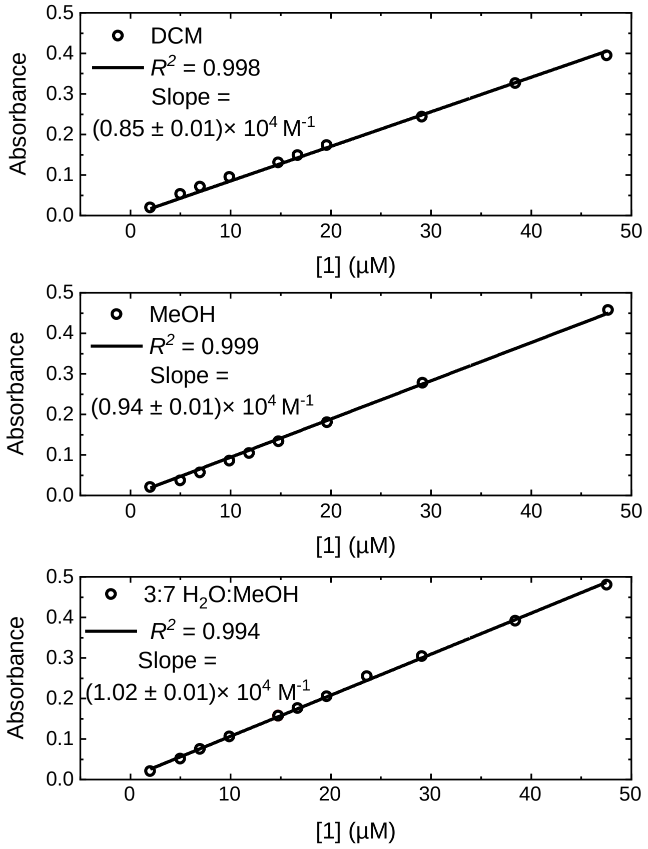

Figure S9. Absorbance of the 0-0 transition set against the concentration of **1**, [1], in the three different solvent systems used.

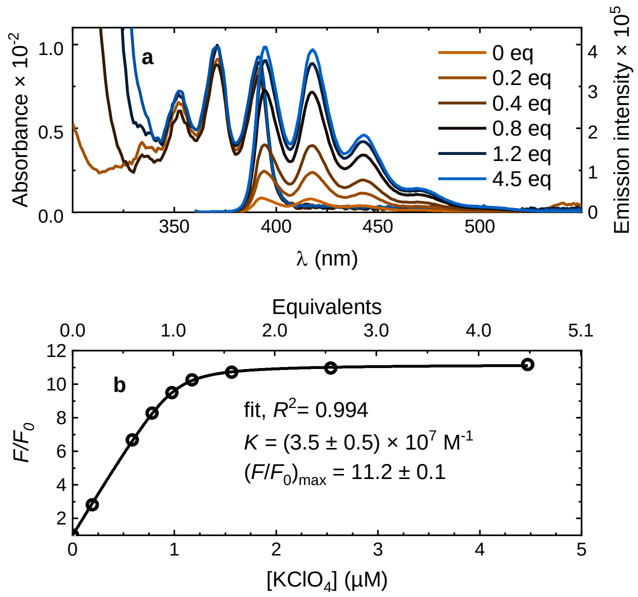

Figure S10: **a)** Absorbance and emission of **1** (1 μM) in DCM with addition of of of 0 to 4.5 mol equivalents of potassium perchlorate ($KClO_4$). **b)** Relative fluorescence intensity, F/F_0, against salt concentration, [$KClO_4$], and binding curve fit to Equation S13.

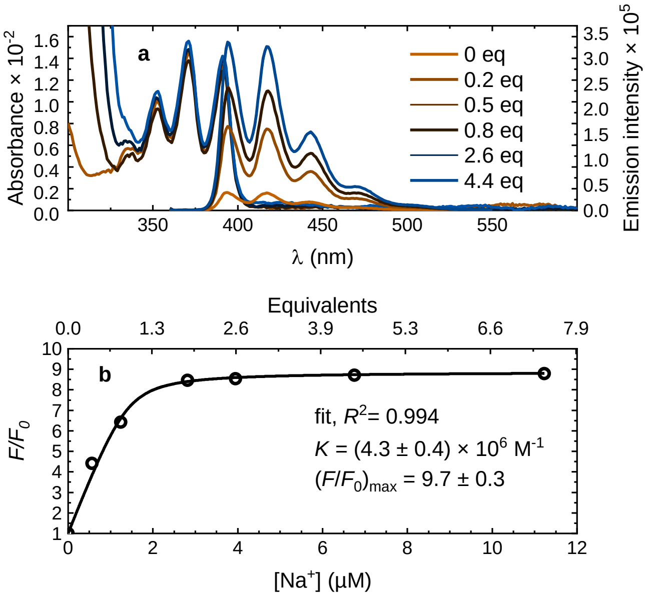

Figure S11: **a)** Absorbance and emission of **1** (1.5 µM) in DCM with addition of of 0 to 4.4 mol equivalents of sodium perchlorate ($NaClO_4$). **b)** Relative fluorescence intensity, F/F_0, against salt concentration, [$NaClO_4$], and binding curve fit to Equation S13.

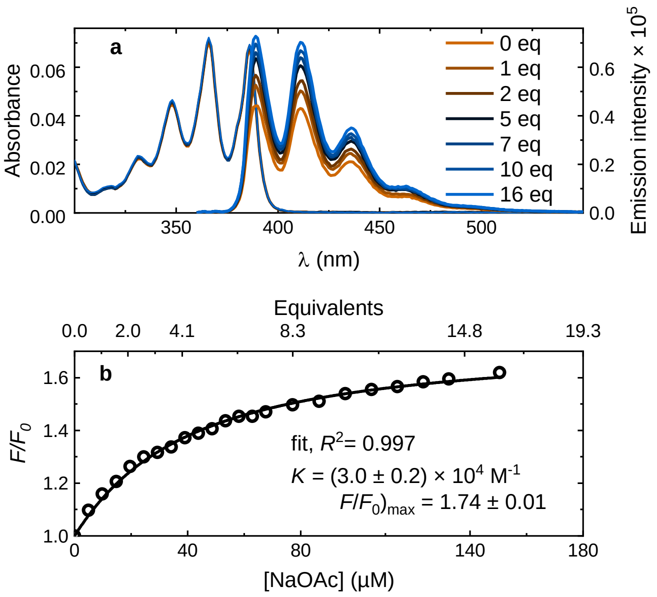

Figure S12: **a)** Absorbance and emission of **1** (9.9 µM) in methanol with addition of of 0 to 16 mol equivalents of sodium acetate (NaOAc). **b)** Relative fluorescence intensity, F/F_0, against salt concentration [NaOAc], and binding curve fit to Equation S13.

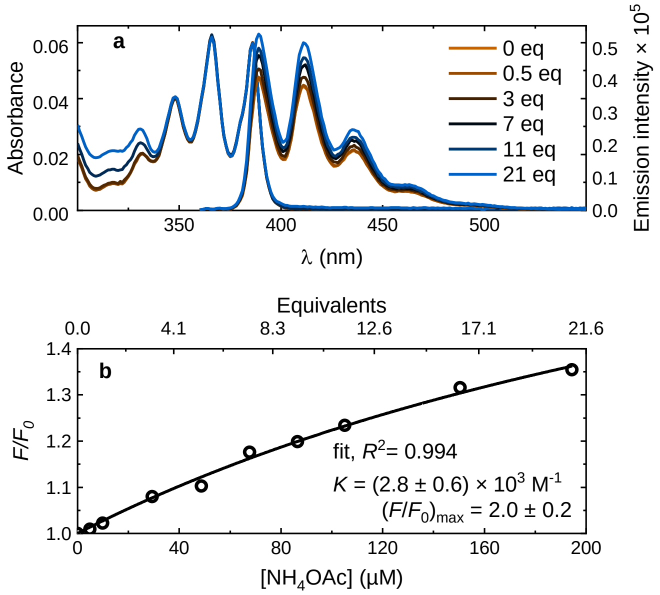

Figure S13: **a)** Absorbance and emission of **1** (9.9 µM) in methanol with addition of of 0 to 21 mol equivalents of ammonium acetate (NH_4OAc). **b)** Relative fluorescence intensity, F/F_0, against salt concentration [NH_4OAc], and binding curve fit to Equation S13.

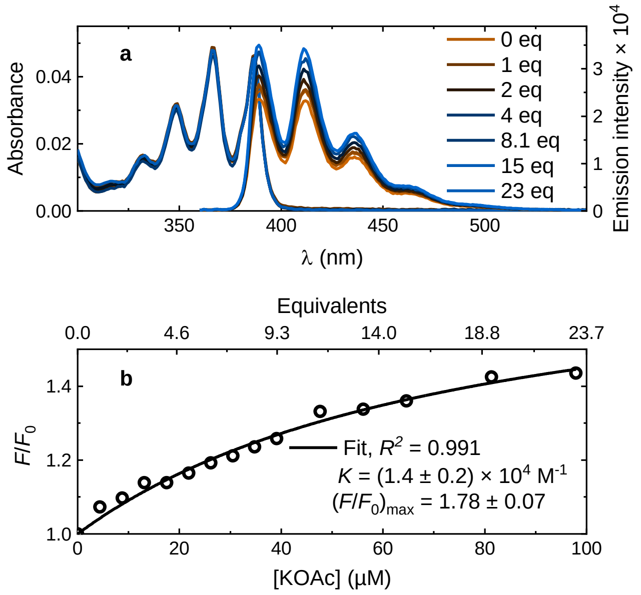

Figure S14: **a)** Absorbance and emission of **1** (4.4 µM) in 3:7 water:methanol with addition of of 0 to 23 mol equivalents of potassium acetate (KOAc). **b)** Relative fluorescence intensity, F/F_0, against salt concentration [KOAc], and binding curve fit to Equation S13.

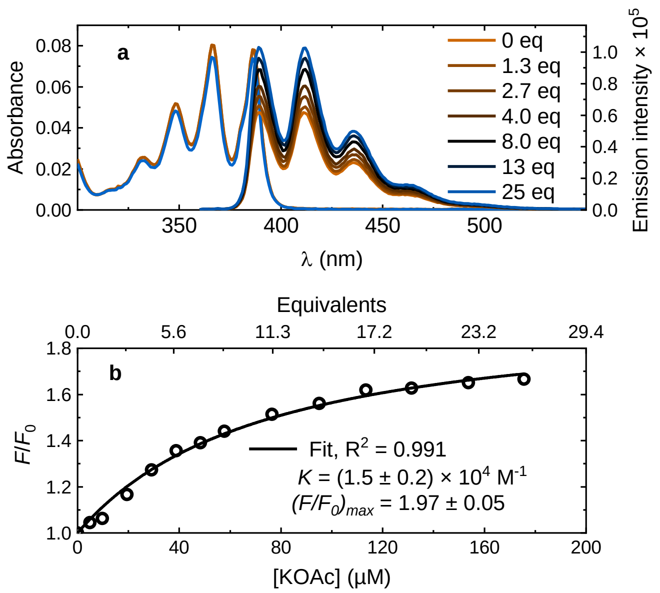

Figure S15: **a)** Absorbance and emission of **1** (7.4 μM) in 3:7 water:methanol with addition of of 0 to 25.2 mol equivalents of potassium acetate (KOAc) and 0 to 2.9 equivalents of $NaClO_4$. **b)** Relative fluorescence intensity, F/F_0, against salt concentration [KOAc], and binding curve fit to Equation S13.

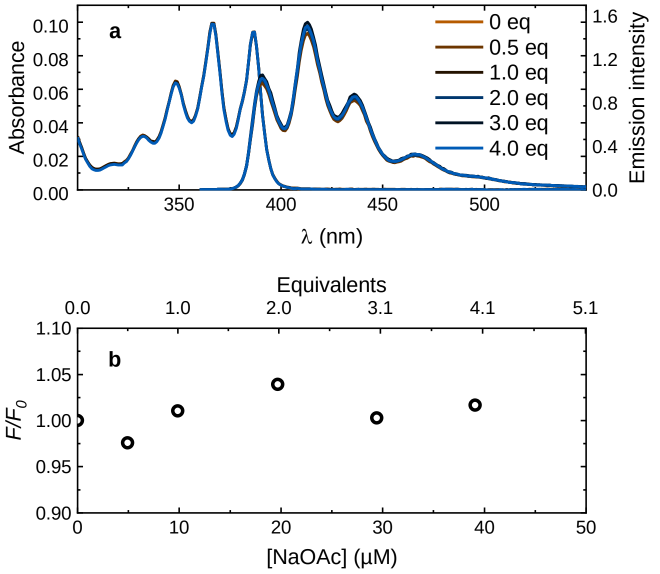

Figure S16: **a)** Absorbance and emission of **1** (9.9 μM) in 3:7 water:methanol with addition of of 0 to 4 mol equivalents of sodium acetate (NaOAc). **b)** Relative fluorescence intensity, F/F_0, against salt concentration [NH_4OAc], and binding curve fit to Equation S13.

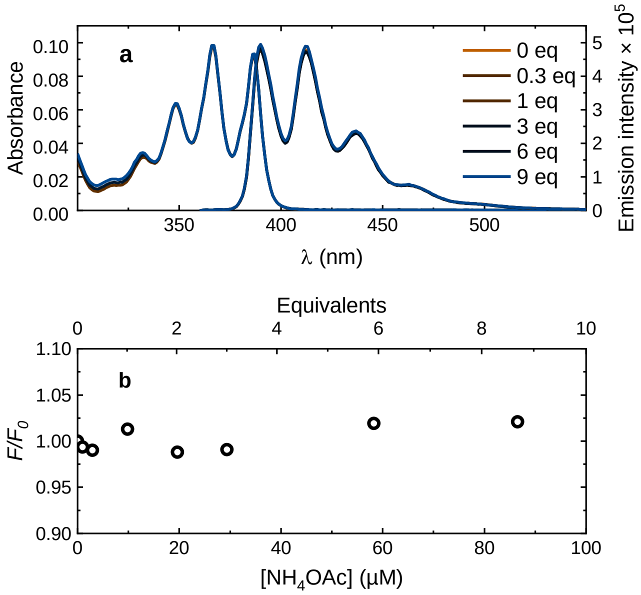

Figure S17: **a)** Absorbance and emission of **1** (9.9 µM) in 3:7 water:methanol with addition of of 0 to 9 mol equivalents of ammonium acetate (NH_4OAc). **b)** Relative fluorescence intensity, F/F_0, against salt concentration [NH_4OAc], and binding curve fit to Equation S13.

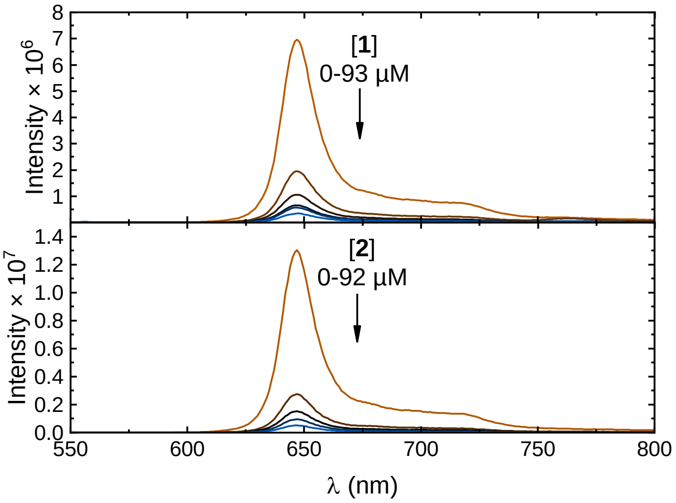

Figure S18: PtOEP emission (5 µM) at increasing quencher concentrations (here molecules **1** and **2**). The emission maximum of PtOEP at 649 nm was used to construct the Stern-Volmer quenching and TET quantum yield curves in Figure 4b in the main text.

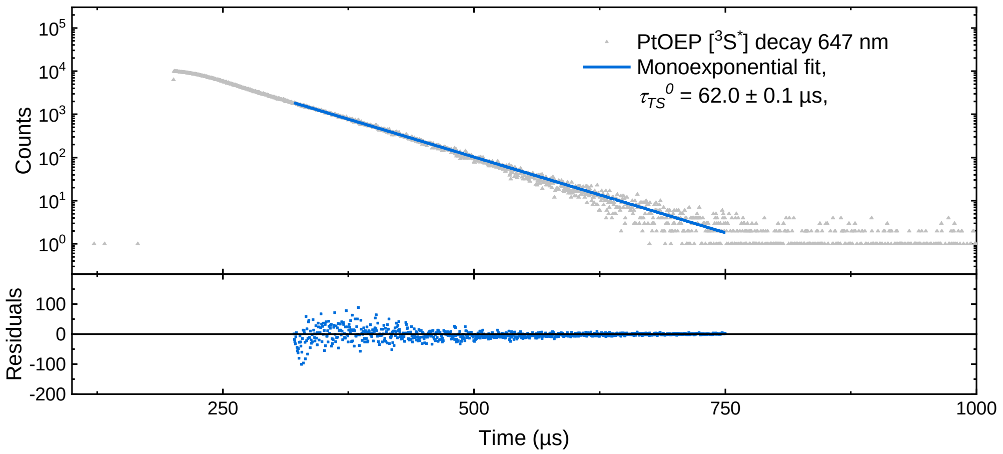

Figure S19: Time-resolved emission of PtOEP (2.5 µM) in 1:1 DCM:MeOH. The decay trace was fit with a single exponential where $\tau_{TS}{}^{0}$ indicates the natural lifetime of the sensitizer PtOEP in the absence of quenching by an annihilator.

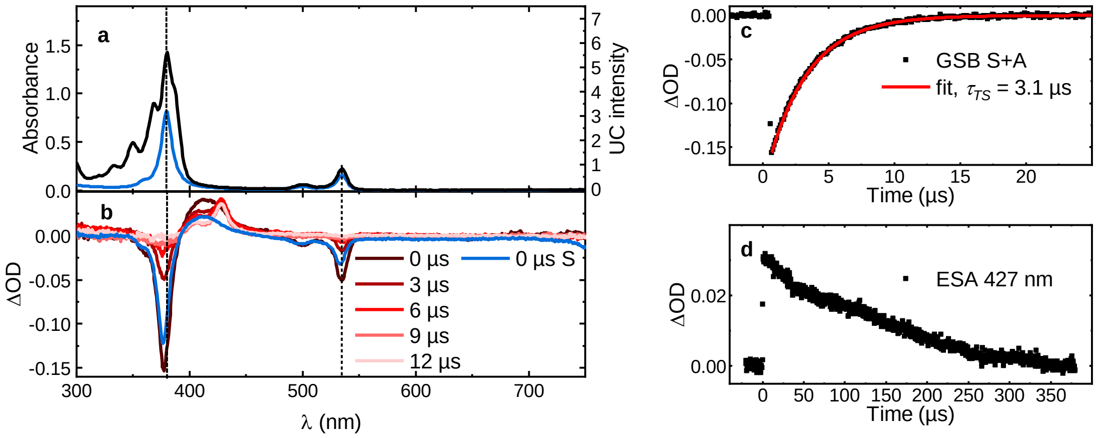

Figure S20: **a)** Absorption spectra of annihilator **1** and sensitizer PtOEP (black, 90 and 5 µM) and and PtOEP (blue, 3 µM) in 1:1 DCM:MeOH. **b)** Transient absorption (TA) emission delay map of the same samples at increasing delay times and PtOEP with 0 delay. **c)** and **d)** Transient kinetics of the ground state bleach (GSB) of the sensitizer at 377.5 nm with the annihilator (**c**) and without the annihilator (**d**).

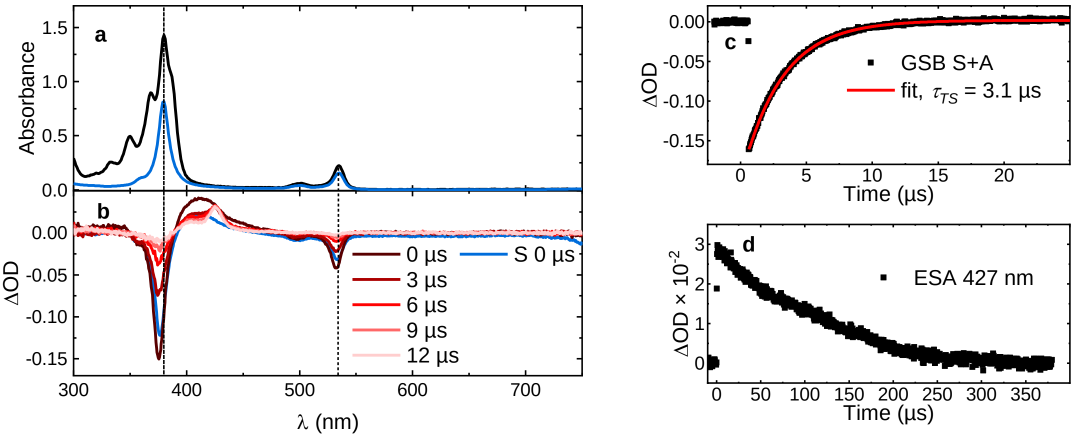

Figure S21: **a)** Absorption spectra of annihilator **1** (77 µM) and sensitizer PtOEP (4.3 µM), with an excess of KOAc (black trace), and PtOEP (blue, 3 µM) in 1:1 DCM:MeOH. **b)** Transient absorption (TA) emission delay map of the same samples at increasing delay times and PtOEP with 0 delay. **c)** and **d)** Transient kinetics of the ground state bleach (GSB) of the sensitizer at 377.5 nm with the annihilator (**c**) and without the annihilator (**d**).

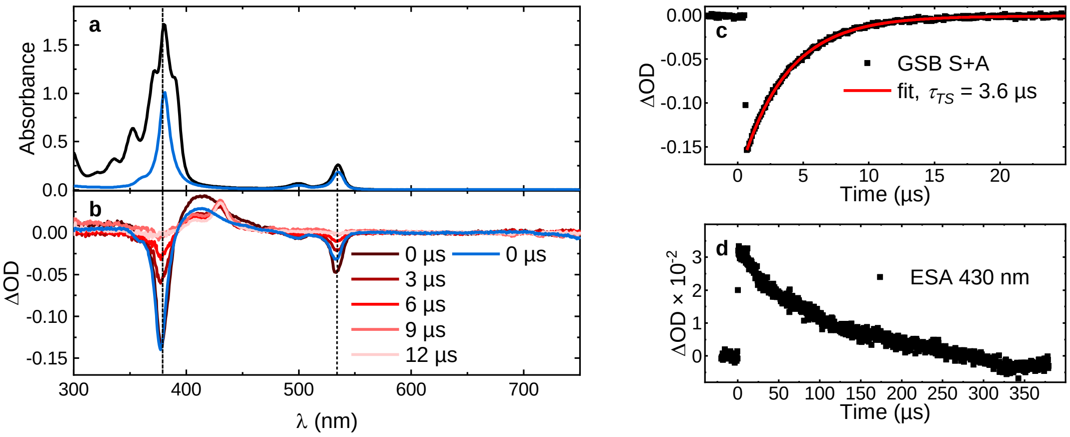

Figure S22: **a)** Absorption spectra of annihilator **1** and sensitizer PtOEP (black, 90 and 5 µM) and and PtOEP (blue, 3 µM) in DCM. **b)** Transient absorption (TA) emission delay map of the same samples at increasing delay times and PtOEP with 0 delay. **c)** and **d)** Transient kinetics of the ground state bleach (GSB) of the sensitizer at 377.5 nm with the annihilator (**c**) and without the annihilator (**d**) fit to an exponential association model.

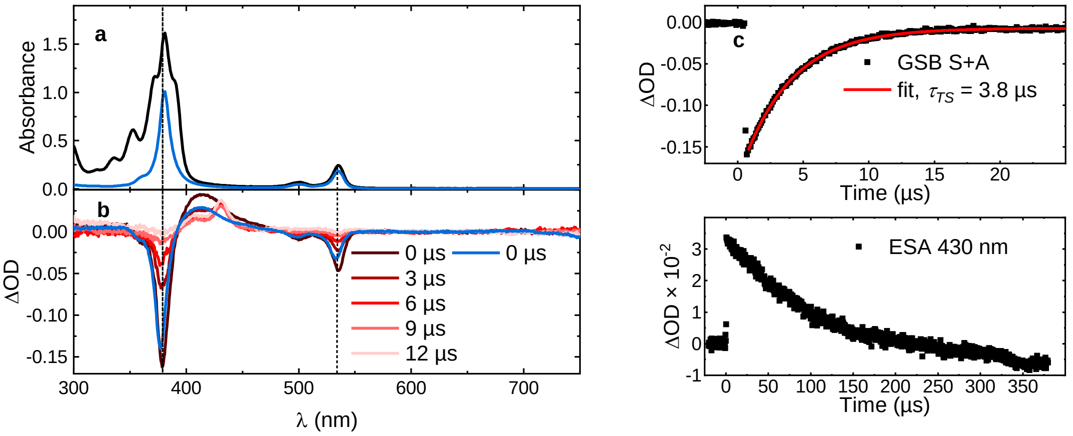

Figure S23: **a)** Absorption spectra of annihilator **1** (84 µM) and sensitizer PtOEP (4.7 µM), with an excess of KOAc (black trace), and PtOEP (blue, 3 µM) in DCM. **b)** Transient absorption (TA) emission delay map of the same samples at increasing delay times and PtOEP with 0 delay. **c)** and **d)** Transient kinetics of the ground state bleach (GSB) of the sensitizer at 377.5 nm with the annihilator (**c**) and without the annihilator (**d**).

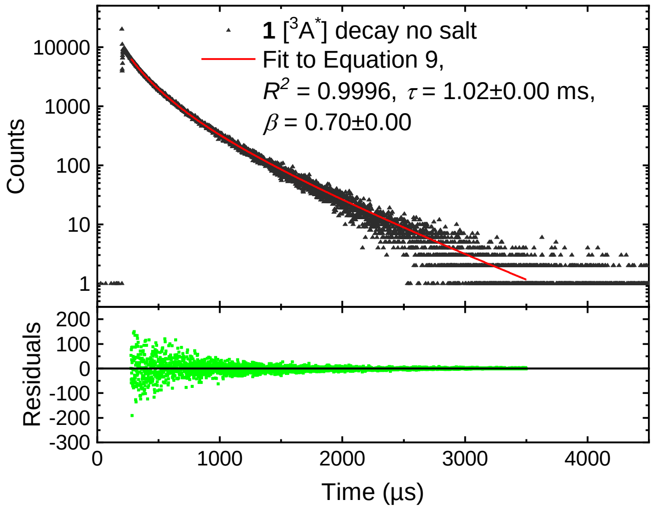

Figure S24: Time-resolved TTA-UC emission of the annihilator in a solution of **1** (45 µM) and PtOEP (2.5 µM) in 1:1 DCM:MeOH. The decay trace was fit to Equation S15 with $n = 2$.

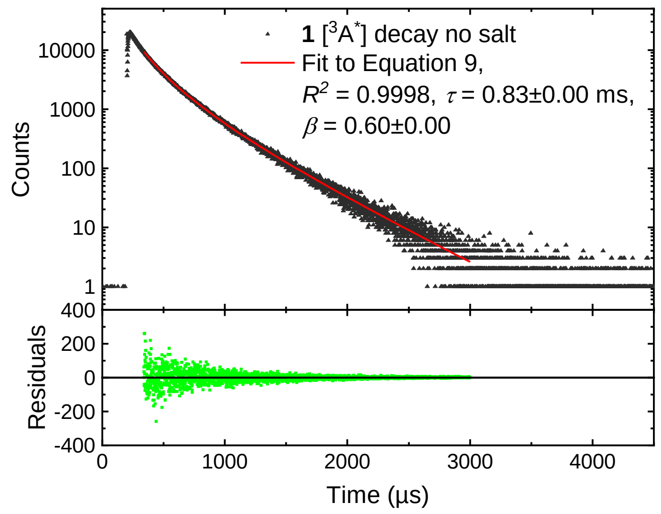

Figure S25: Time-resolved TTA-UC emission of the annihilator in a solution of **1** (45 µM) and PtOEP (3 µM) in 1:1 DCM:MeOH. The decay trace was fit to Equation S15 with *n* = 2.

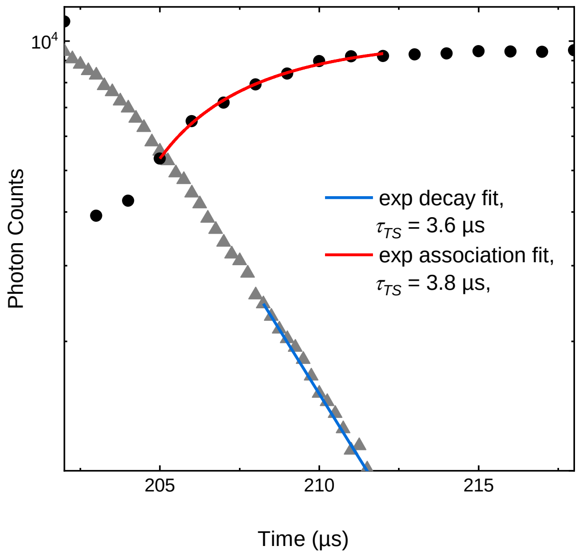

Figure S26: Start of time-resolved emission of **1** (415 nm, ●), and sensitizer decay (647 nm, ▲) in a solution of **1** (45 µM) and PtOEP (2.5 µM) in 1:1 DCM:MeOH. The rise of **1** has been fit to an exponential association model (red) and the sensitizer decay is fit to the double exponential in Figure S36.

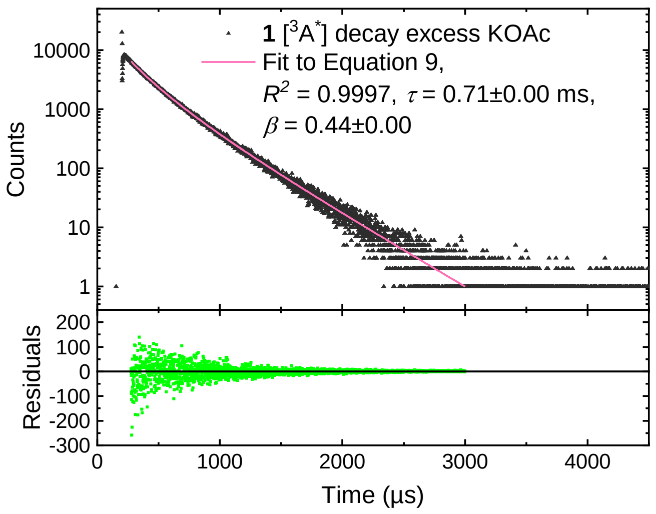

Figure S27: Time-resolved TTA-UC emission of the annihilator in a solution of **1** (39 µM), PtOEP (2.2 µM) and excess KOAc in 1:1 DCM:MeOH. The decay trace was fit to Equation S15 with $n = 2$.

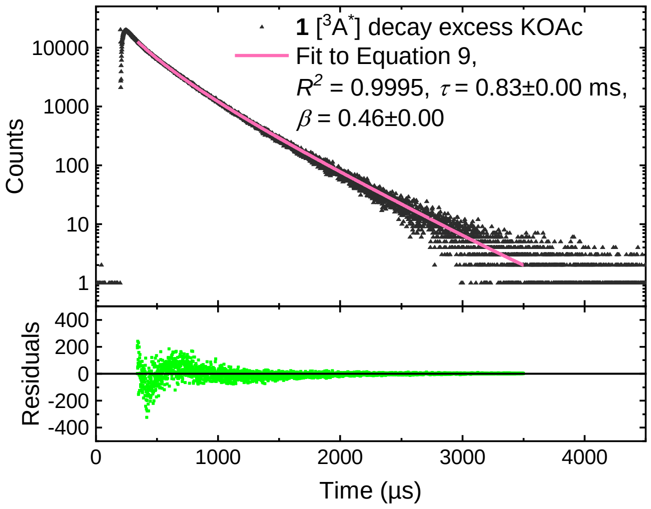

Figure S28: Time-resolved TTA-UC emission of the annihilator in a solution of **1** (45 µM), PtOEP (3 µM) and excess KOAc in 1:1 DCM:MeOH. The decay trace was fit to Equation S15 $n = 2$.

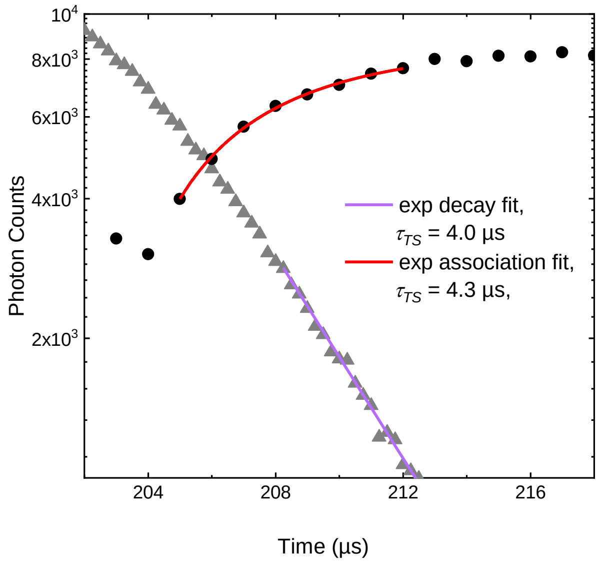

Figure S29: Start of time-resolved emission of **1** (415 nm, ●), and sensitizer decay (647 nm, ▲) in a solution of **1** (45 µM) and PtOEP (2.5 µM), with an excess of KOAc in 1:1 DCM:MeOH. The rise of **1** has been fit to an exponential association model (red) and the sensitizer decay is fit to the double exponential in Figure S37.

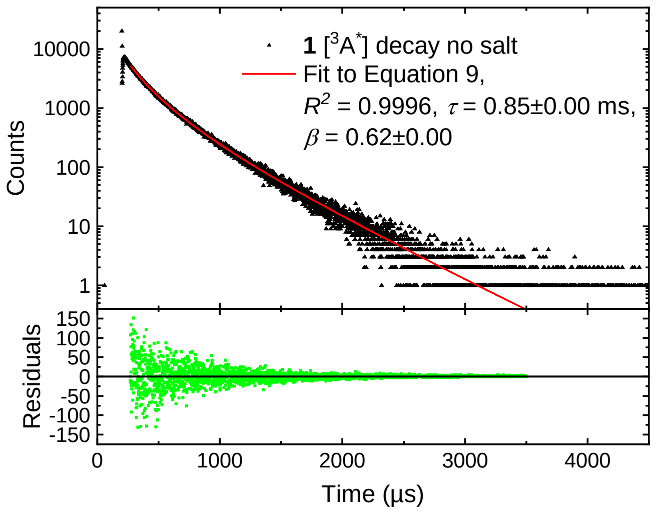

Figure S30: Time-resolved TTA-UC emission of the annihilator in a solution of **1** (45 µM) and PtOEP (2.5 µM) in DCM. The decay trace was fit to Equation S15 with $n = 2$.

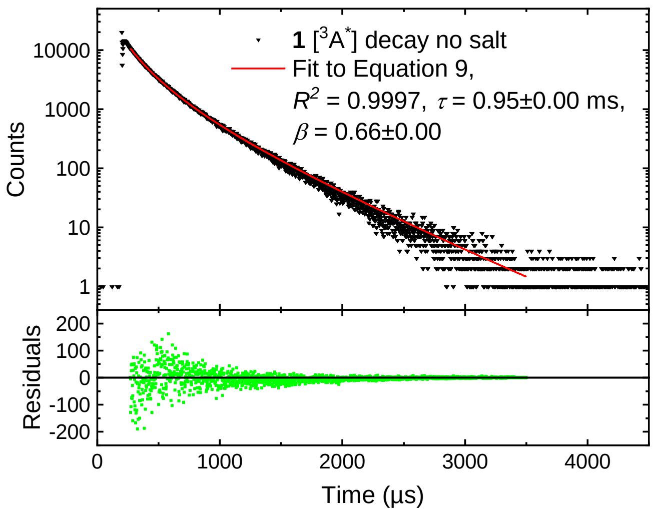

Figure S31: Time-resolved TTA-UC emission of the annihilator in a solution of **1** (45 µM) and PtOEP (2.5 µM) in DCM. The decay trace was fit to Equation S15 with n = 2.

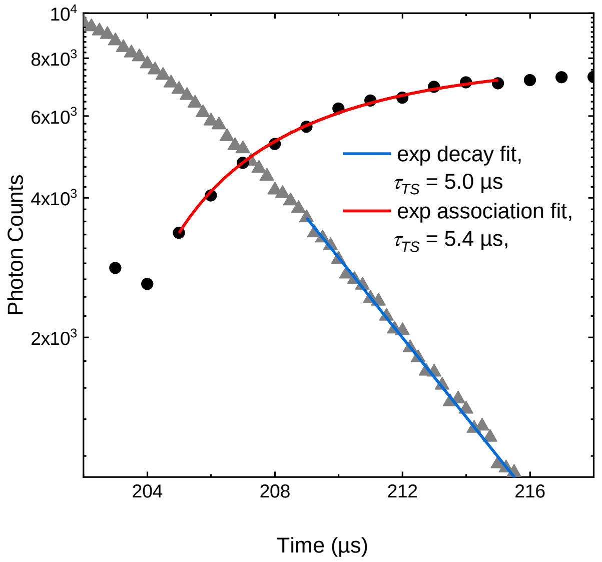

Figure S32: Start of time-resolved emission of **1** (415 nm, ●), and sensitizer decay (647 nm, ▲) in a solution of **1** (45 µM) and PtOEP (2.5 µM) in DCM. The rise of **1** has been fit to an exponential association model (red) and the sensitizer decay is fit to the double exponential in Figure S38.

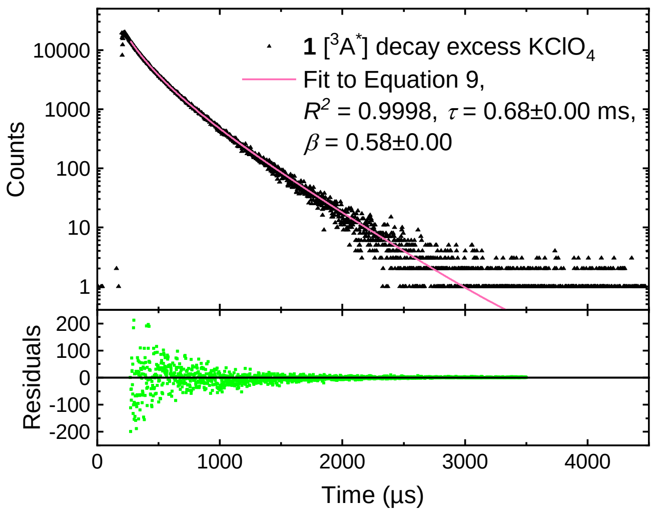

Figure S33: Time-resolved TTA-UC emission of **1** in a solution of **1** (45 µM), PtOEP (5 µM), and excess $KClO_4$ in DCM. The decay trace was fit to Equation S15 with *n* = 2.

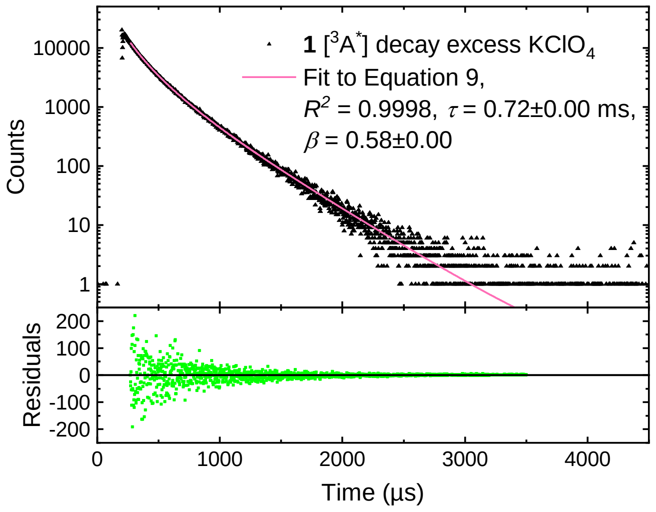

Figure S34: Time-resolved TTA-UC emission of **1** in a solution of **1** (45 µM), PtOEP (5 µM), and excess $KClO_4$ in DCM. The decay trace was fit to Equation S15 with *n* = 2.

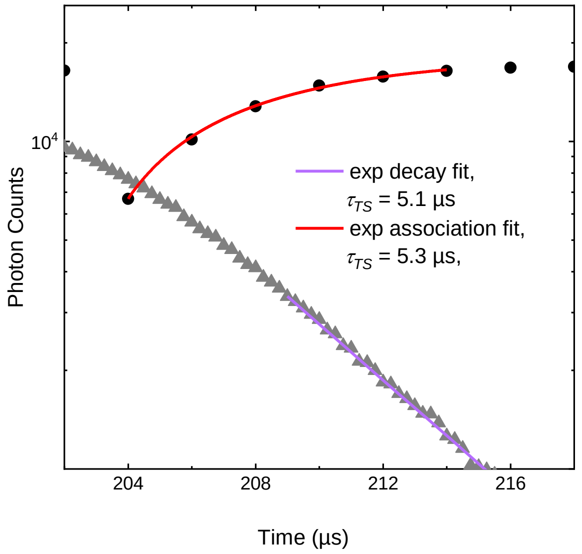

Figure S35: Start of time-resolved emission of **1** (415 nm, ●), and sensitizer decay (647 nm, ▲) in a solution of **1** (45 µM) and PtOEP (2.5 µM) in DCM with an excess of $KClO_4$. The rise of **1** has been fit to an exponential association model (red) and the sensitizer decay is fit to the double exponential in Figure S40.

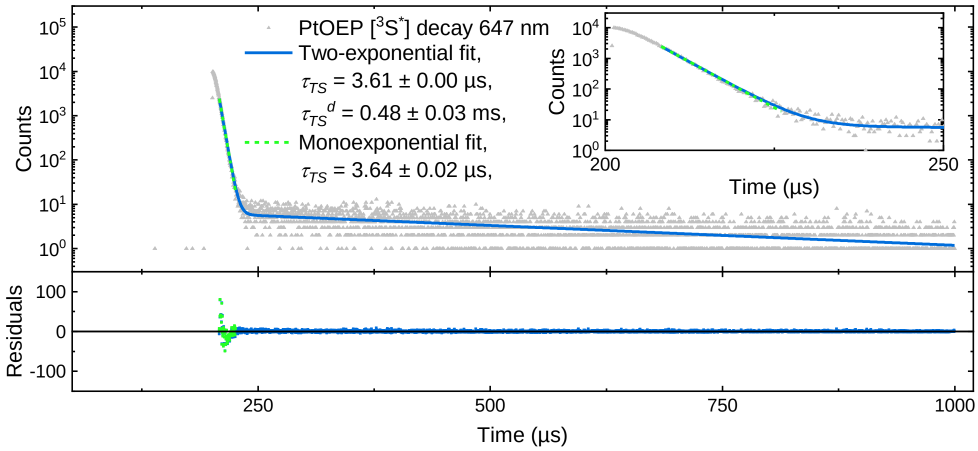

Figure S36: Time-resolved emission of the sensitizer (PtOEP) in a solution of **1** (45 µM) and PtOEP (2.5 µM) in 1:1 DCM:MeOH. The decay trace was fit with a double exponential where $\tau_{TS}{}^{d}$ indicates the approximate lifetime of the second, slow and weak component. The initial rapid decay has also been fitted with a single exponential for comparison (inset).

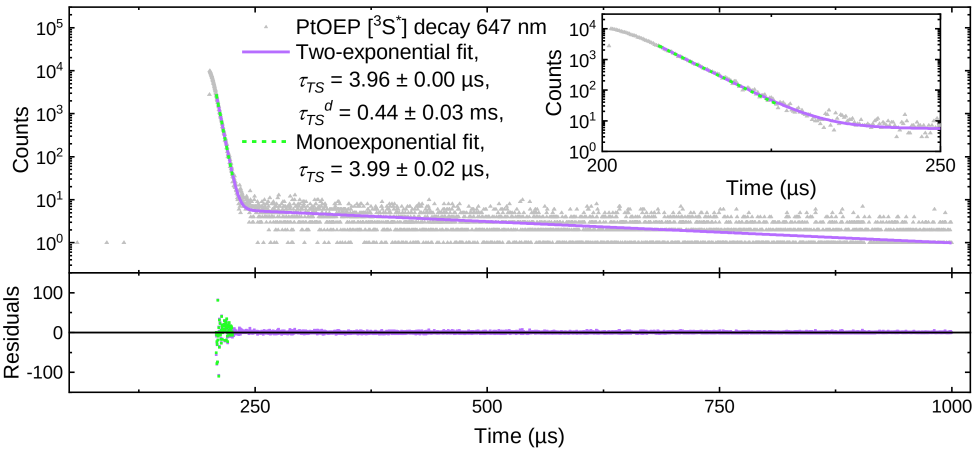

Figure S37: Time-resolved emission of the sensitizer (PtOEP) in a solution of **1** (45 µM), PtOEP (2.5 µM) and excess KOAc in 1:1 DCM:MeOH. The decay trace was fit with a double exponential where $\tau_{TS}{}^{d}$ indicates the approximate lifetime of the second, slow and weak component. The initial rapid decay has also been fitted with a single exponential for comparison (inset).

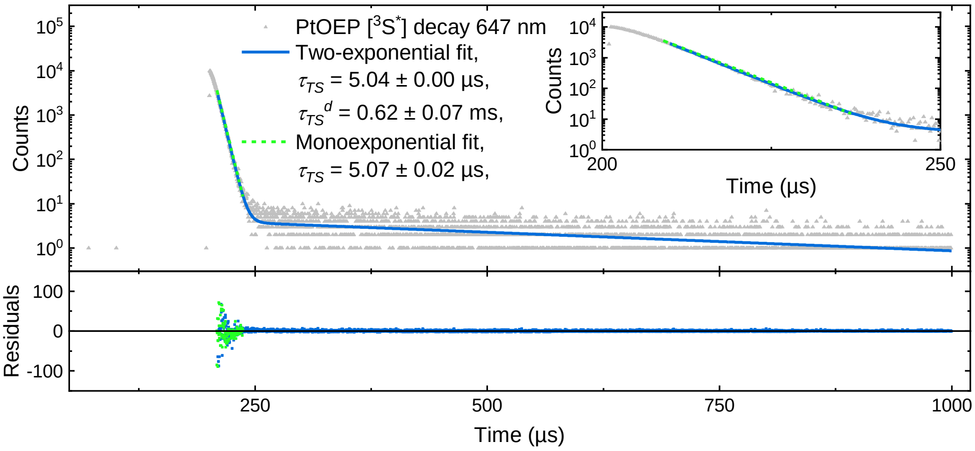

Figure S38: Time-resolved emission of the sensitizer (PtOEP) in a solution of **1** (45 µM) and PtOEP (2.5 µM) in DCM. The decay trace was fit with a double exponential where $\tau_{TS}{}^{d}$ indicates the approximate lifetime of the second, slow and weak component. The initial rapid decay has also been fitted with a single exponential for comparison (inset).

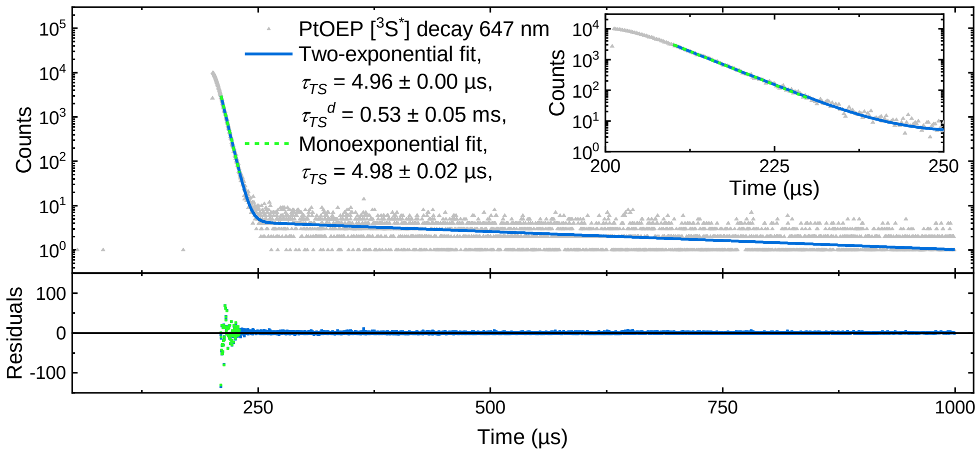

Figure S39: Time-resolved emission of the sensitizer (PtOEP) in a solution of **1** (45 µM) and PtOEP (2.5 µM) in DCM. The decay trace was fit with a double exponential where $\tau_{TS}{}^{d}$ indicates the approximate lifetime of the second, slow and weak component. The initial rapid decay has also been fitted with a single exponential for comparison (inset).

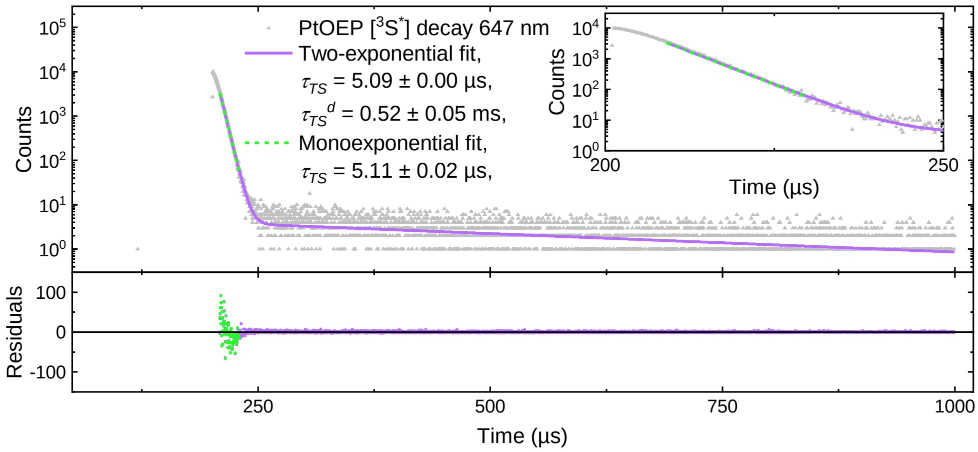

Figure S40: Time-resolved emission of the sensitizer (PtOEP) in a solution of **1** (45 µM), PtOEP (2.5 µM) and excess $KClO_4$ in DCM. The decay trace was fit with a double exponential where $\tau_{TS}{}^{d}$ indicates the approximate lifetime of the second, slow and weak component. The initial rapid decay has also been fitted with a single exponential for comparison (inset).

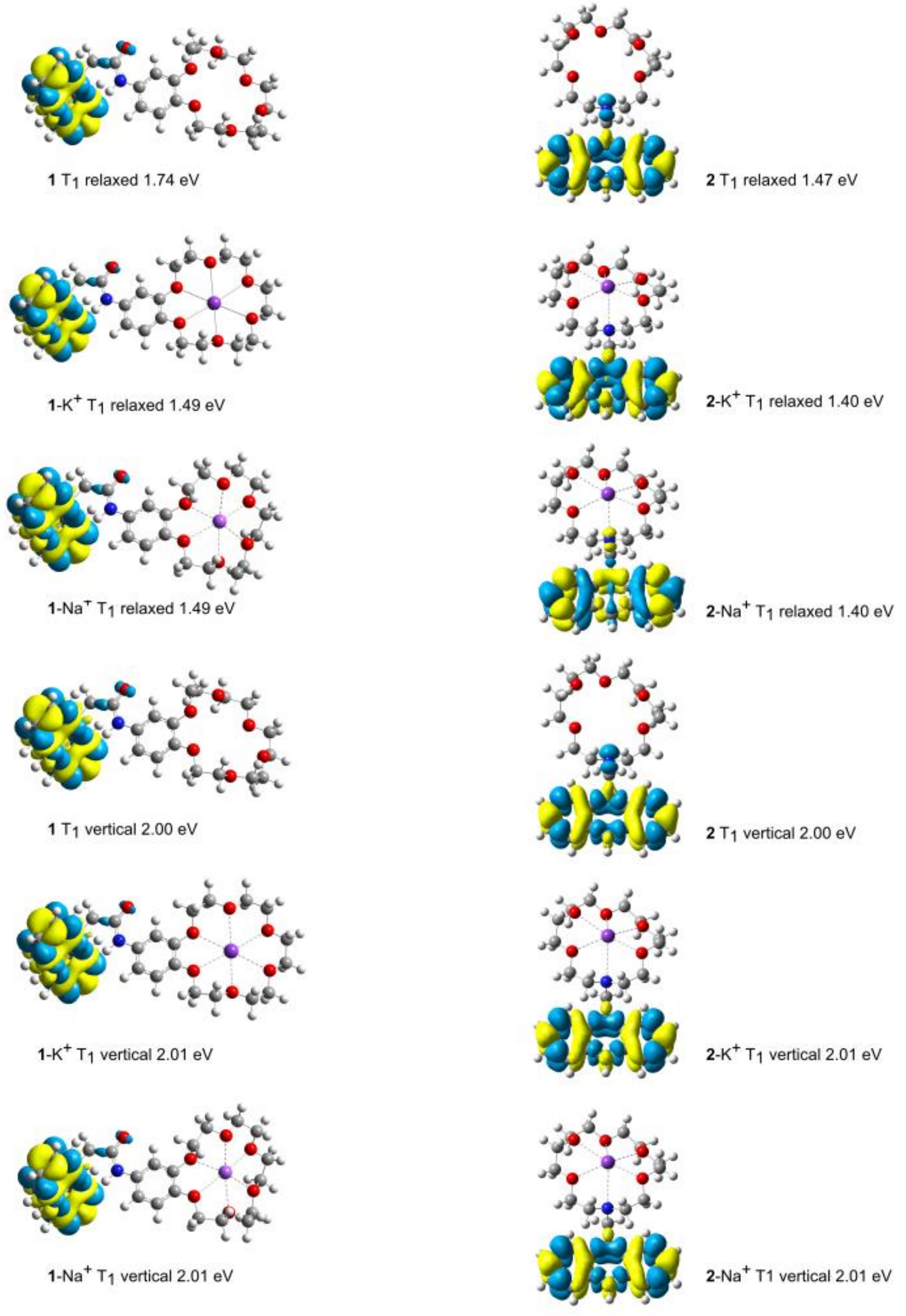

Figure S41: Electron density difference maps of the lowest triplet (T_1) state of molecule 1 and 2 in the free, and. K^+- and Na^+ complexing forms.

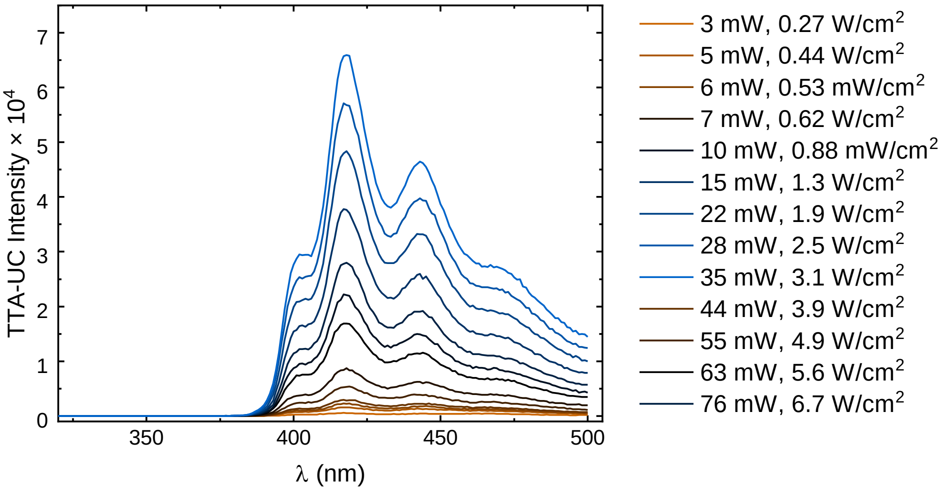

Figure S42: Upconversion spectra of **1** (90 μM) sensitized by PtOEP (5 μM) at excitation powers of 3-76 mW, corresponding to 0.27-6.7 W/cm^2 excitation intensities.

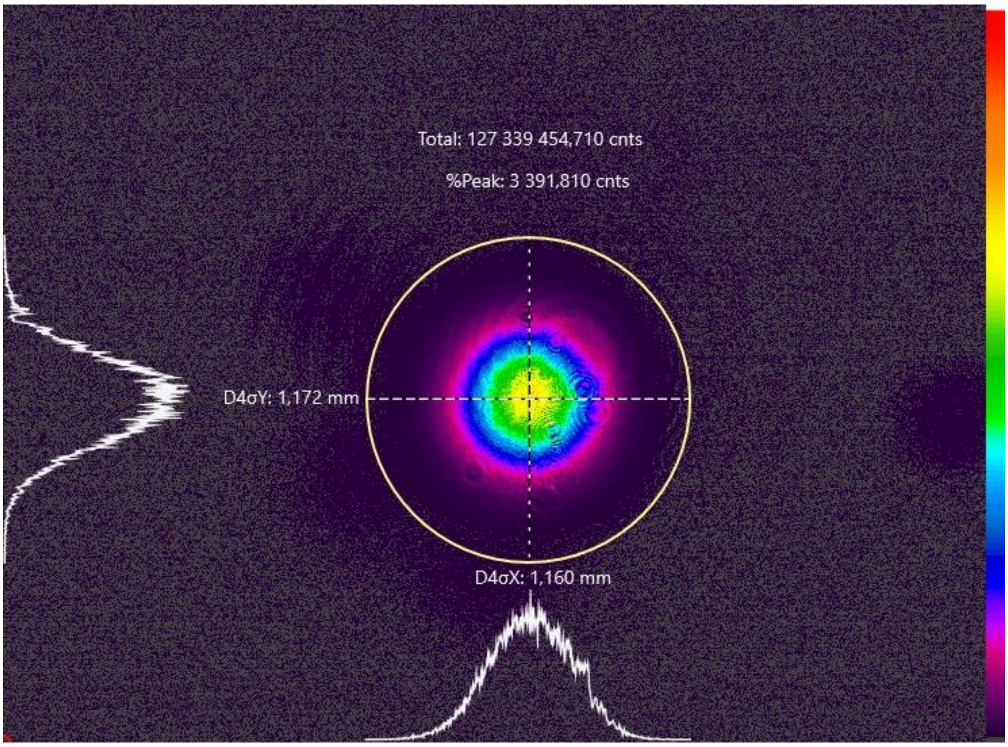

Figure S43: Beam profile of the cw laser diode (OBIS 532LS, 100 mW power) monitored with an Ophir SP932U beam profiler. The camera was placed in the sample compartment right after the location of the cuvette in the beam path used in upconversion experiments. D4σX/Y refers to a beam width of 4 standard derivations from the center of the Gaussian beam, in accordance with ISO guidelines.[1]

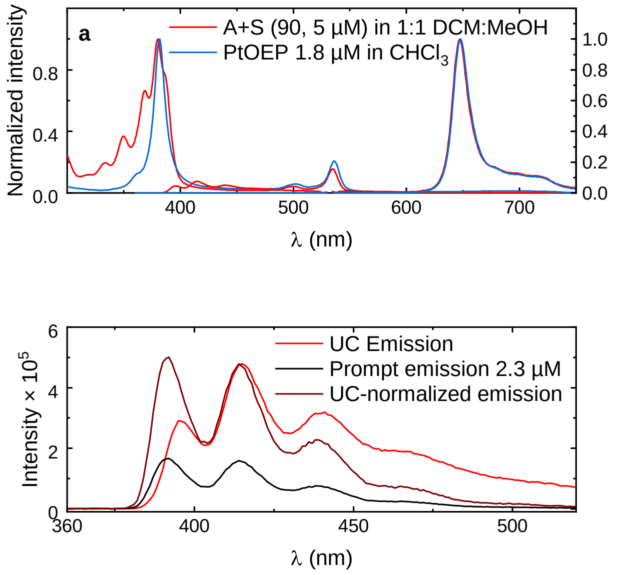

Figure S44: **a)** Normalized absorption and emission of the sensitizer PtOEP (blue) in $CHCl_3$, and the annihilator, **1**, with sensitizer (red) in 1:1 DCM:MeOH. **b)** Prompt and upconverted fluorescence emission spectra of **1** used to correct for the inner filter effect. The ratio of 0-0 peaks of the UC emission to the normalized spectrum in brown (UC normalized emission 1 µM) was used to determine the outcoupling efficiency, Φ_{exp}.

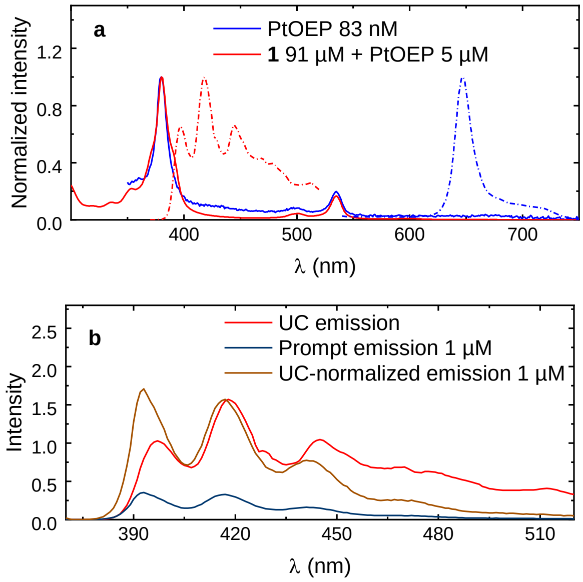

Figure S45: **a)** Normalized absorption and emission of the sensitizer PtOEP (blue), and the annihilator **1** with sensitizer (red) in DCM. **b)** Prompt and upconverted fluorescence emission spectra of **1** used to correct for the inner filter effect. The ratio of 0-0 peaks of the UC emission to the normalized spectrum in brown (UC normalized emission 1 µM) was used to determine the outcoupling efficiency, Φ_{exp}.

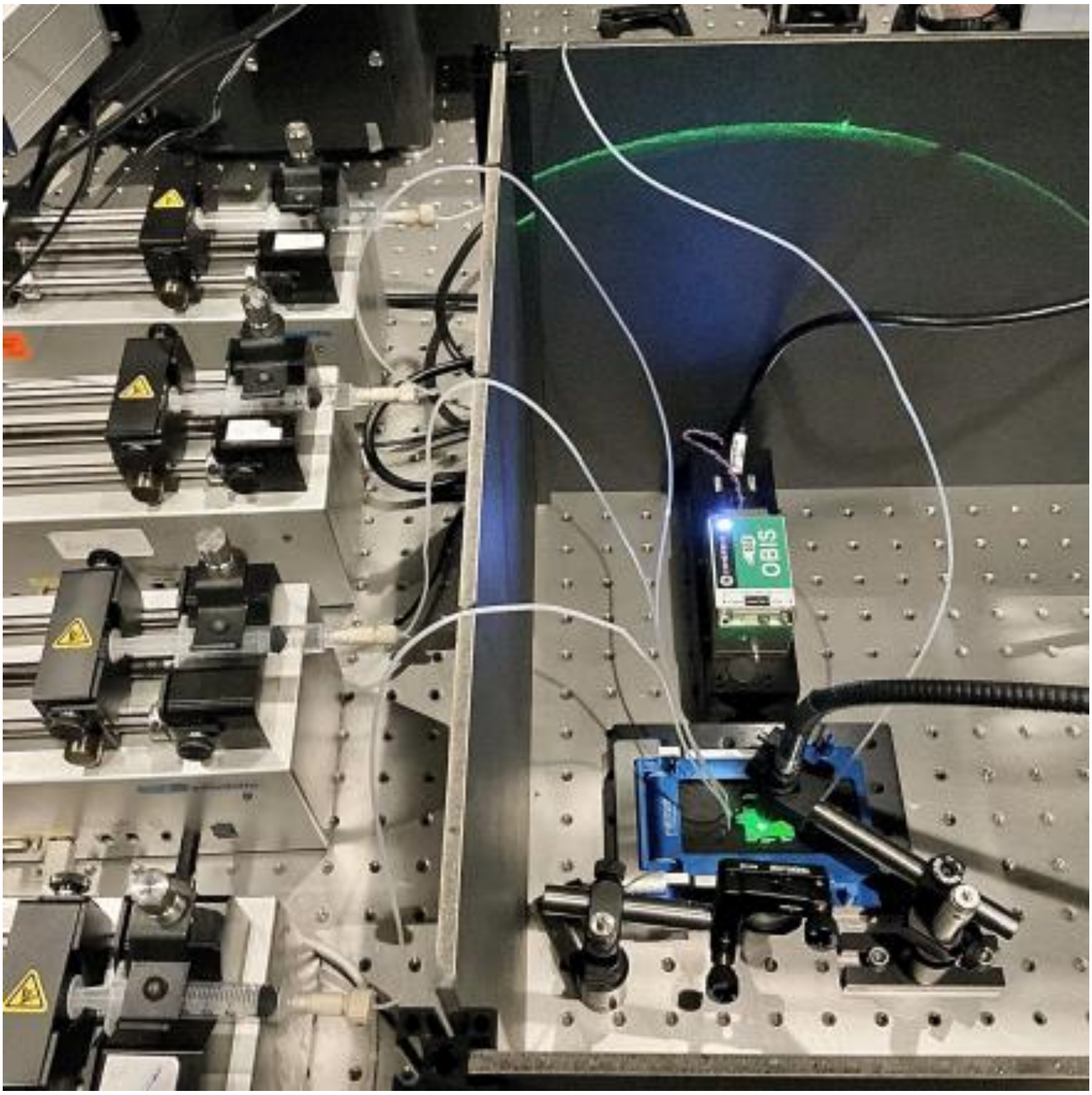

Figure S46: Microfluidic device setup, including syringes with pumps (left), microfluidic glass chip in holder, laser diode (532 nm), reflective mirror and liquid lightguide connected to a lens 90° to the chip surface.

6. Supplementary Tables

Table S1: TD-DFT properties of the excited states of molecular sensors 1 and 2 in the free and K^+-, or Na^+-complexing form, calculated at the TDA-B3LYP/6-31+G(d,p) level. Energies are denoted ΔE_{gap} for the energy gap between orbitals of the most prominent transition, ΔE_V for vertical, ΔE_{Ad} for adiabatic, and ΔE_{Rel} for relaxed transition energies. ΔE_{Abs}^{exp} and ΔE_{Em}^{exp} indicate the experimental absorption and emission energies for the LE state in MeOH (Fig. S6).

Property/ Species	State	ΔE_{gap}^{a} /eV	ΔE_V /eV	ΔE_{Ad}/ eV	ΔE_{Rel}/ eV	ΔE_{Abs}^{exp}/ eV	ΔE_{Em}^{exp}/ eV	Oscillator strength[a]	CI coefficients[a]
1	CSS	2.28	2.55	2.13	1.72	N/A	N/A	0.0000	HOMO → LUMO: 0.71
	LES	3.03	3.30	3.14	2.99	3.21	3.18	0.1615	HOMO-1 → LUMO: 0.68
	T_1	2.90	2.00	1.73	1.47	-	-	0.0000	HOMO-1 → LUMO: 0.69
**1**K^+	CSS	3.28	3.87	3.28	2.77	N/A	N/A	0.0041	HOMO-1 → LUMO: 0.67<br>HOMO → LUMO: -0.24
	LES	3.26	3.33	3.15	3.02	3.21	3.18	0.1525	HOMO → LUMO: 0.68<br>HOMO-2 → LUMO+7: -0.11
	T_1	2.92	2.01	1.75	1.49	-	-	0.0000	HOMO → LUMO: 0.63<br>HOMO-3 → LUMO+5: 0.16<br>HOMO-2 → LUMO+3: 0.12<br>HOMO-6 → LUMO+7: 0.11<br>HOMO-5 → LUMO+7: 0.10
**1**Na^+	CSS	-	3.90	-	3.00	N/A	N/A	0.0006	-
	LES	3.05	3.33	3.16	3.02	3.21	3.18	0.1516	HOMO → LUMO: 0.68<br>HOMO-2 → LUMO+7: -0.11
	T_1			1.73	1.49	-	-	0.0000	HOMO → LUMO: 0.70
2	CSS	2.28	2.94	1.91	1.62	N/A	N/A	0.0002	HOMO → LUMO: 0.71
	LE	3.39	3.39	3.22	3.03	3.19	3.15	0.1060	HOMO → LUMO: 0.57<br>HOMO-1 → LUMO: -0.38
	T_1	2.87	2.00	1.73	1.47	-	-	0.0000	HOMO → LUMO: 0.68<br>HOMO-1 → LUMO: -0.17
**2**K^+	CSS	-	4.13	-	-	N/A	N/A	-	-
	LES	3.02	3.32	3.15	2.98	3.19	3.15		HOMO → LUMO: 0.68<br>HOMO-1 → LUMO+2: 0.11
	T_1	2.86	2.01	1.74	1.40	-	-	0.0000	HOMO → LUMO: 0.70
**2**Na^+	CSS	-	4.13	-	-	N/A	N/A	-	-
	LES	3.02	3.19	3.15	2.98	3.19	3.15	0.1426	HOMO → LUMO: 0.68<br>HOMO-1 → LUMO+2: 0.11
	T_1	3.44	2.01	1.74	1.40	-	-	0.0000	HOMO → LUMO: 0.69-

[a]Values for the geometry optimized excited state.

7. References

(1) (ISO), I. O. f. S. *ISO 11146-1:2021 Lasers and laser-related equipment — Test methods for laser beam widths, divergence angles and beam propagation ratios*; 2021.

(2) Brouwer, A. M. Standards for photoluminescence quantum yield measurements in solution (IUPAC Technical Report). *Pure and Applied Chemistry* **2011**, *83* (12), 2213-2228. DOI: 10.1351/PAC-REP-10-09-31.

(3) Bansal, A. K.; Holzer, W.; Penzkofer, A.; Tsuboi, T. Absorption and emission spectroscopic characterization of platinum-octaethyl-porphyrin (PtOEP). *Chem. Phys.* **2006**, *330* (1), 118-129. DOI: https://doi.org/10.1016/j.chemphys.2006.08.002.

(4) Vosko, S. H.; Wilk, L.; Nusair, M. Accurate spin-dependent electron liquid correlation energies for local spin density calculations: a critical analysis. *Can. J. Phys.* **1980**, *58* (8), 1200-1211. DOI: 10.1139/p80-159.

(5) Lee, C.; Yang, W.; Parr, R. G. Development of the Colle-Salvetti correlation-energy formula into a functional of the electron density. *Phys. Rev. B.* **1988**, *37* (2), 785-789. DOI: 10.1103/PhysRevB.37.785.

(6) Becke, A. D. Density-functional thermochemistry. III. The role of exact exchange. *J. Chem. Phys.* **1993**, *98* (7), 5648-5652. DOI: 10.1063/1.464913.

(7) Stephens, P. J.; Devlin, F. J.; Chabalowski, C. F.; Frisch, M. J. Ab Initio Calculation of Vibrational Absorption and Circular Dichroism Spectra Using Density Functional Force Fields. *J. Phys. Chem.* **1994**, *98* (45), 11623-11627. DOI: 10.1021/j100096a001.

(8) Frisch, M. J.; Trucks, G. W.; Schlegel, H. B.; Scuseria, G. E.; Robb, M. A.; Cheeseman, J. R.; Scalmani, G.; Barone, V.; Petersson, G. A.; Nakatsuji, H.; et al. Gaussian 16 Rev. A.03. **2016**.

(9) Gromov, E. V.; Burghardt, I.; Köppel, H.; Cederbaum, L. S. Impact of Sulfur vs Oxygen on the Low-Lying Excited States of trans-p-Coumaric Acid and trans-p-Coumaric Thio Acid. *J. Phys. Chem. A.* **2005**, *109* (20), 4623-4631. DOI: 10.1021/jp0447791.

(10) Grabowski, D.; Alef, S.; Becker, S.; Müller, U.; Schnakenburg, G.; Höger, S. Condensation of pyrylium salts with mixed anhydrides: aryl ethers, aryl amines and sterically congested aromatics. *Organic Chemistry Frontiers* **2022**, *9* (2), 294-298. DOI: 10.1039/D1QO01419F.

(11) Bissell, R. A.; Calle, E.; de Silva, A. P.; de Silva, S. A.; Gunaratne, H. Q. N.; Habib-Jiwan, J.-L.; Peiris, S. L. A.; Rupasinghe, R. A. D. D.; Shantha, T. K.; Samarasinghe, D.; et al. Luminescence and charge transfer. Part 2. Aminomethyl anthracene derivatives as fluorescent PET (photoinduced electron transfer) sensors for protons. *J. Chem. Soc., Perkin Trans.* **1992**, (9), 1559-1564. DOI: 10.1039/P29920001559.

(12) Saltiel, J.; March, G. R.; Smothers, W. K.; Stout, S. A.; Charlton, J. L. Spin-statistical factor in the triplet-triplet annihilation of anthracene triplets. *J. Am. Chem. Soc.* **1981**, *103* (24), 7159-7164. DOI: 10.1021/ja00414a020.

(13) Bossanyi, D. G.; Sasaki, Y.; Wang, S.; Chekulaev, D.; Kimizuka, N.; Yanai, N.; Clark, J. Spin Statistics for Triplet–Triplet Annihilation Upconversion: Exchange Coupling, Intermolecular Orientation, and Reverse Intersystem Crossing. *JACS Au* **2021**, *1* (12), 2188-2201. DOI: 10.1021/jacsau.1c00322.

(14) Carrod, A. J.; Gray, V.; Börjesson, K. Recent advances in triplet–triplet annihilation upconversion and singlet fission, towards solar energy applications. *Energy Environ. Sci.* **2022**, *15* (12), 4982-5016. DOI: 10.1039/D2EE01600A.

(15) Edhborg, F.; Olesund, A.; Albinsson, B. Best practice in determining key photophysical parameters in triplet–triplet annihilation photon upconversion. *Photochem. Photobiol. Sci.* **2022**, *21* (7), 1143-1158.

(16) Rehm, D.; Weller, A. Kinetics of Fluorescence Quenching by Electron and H-Atom Transfer. *Isr. J. Chem.* **1970**, *8* (2), 259-271. DOI: https://doi.org/10.1002/ijch.197000029.

(17) Weller, A. Photoinduced Electron Transfer in Solution: Exciplex and Radical Ion Pair Formation Free Enthalpies and their Solvent Dependence. *Z. Phys. Chem.* **1982**, *133* (1), 93-98. DOI: doi:10.1524/zpch.1982.133.1.093 (acccessed 2024-05-23).

(18) Chibisov, A. K. Electron Transfer in Photochemical Reactions. *Russ. Chem. Rev.* **1981**, *50* (7), 615. DOI: 10.1070/RC1981v050n07ABEH002656.
